# Supplementary figures and images for: The evolutionary origins and ancestral features of septins
Source: Front Cell Dev Biol. 2024 Jun 26;12:1406966. doi: 10.3389/fcell.2024.1406966 (PMC11238149; doi:10.3389/fcell.2024.1406966)

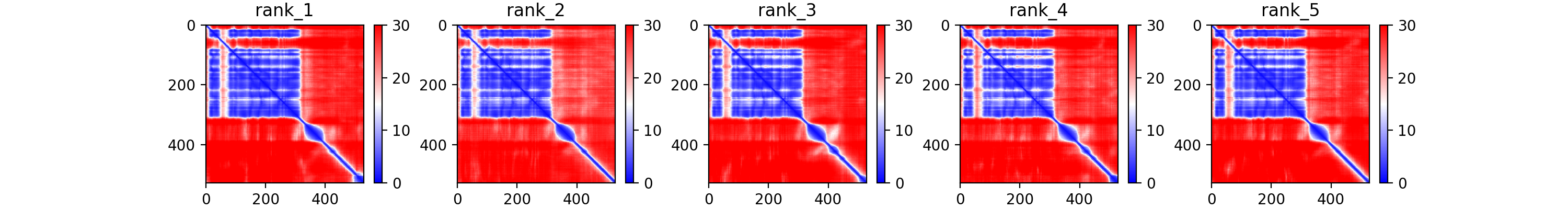

Supplement: Supplementary file 1 [file DataSheet3.zip › Delic_Shuman_Supp_File6/AncGroup3_2f3f8.result/AncGroup3_2f3f8_PAE.png]

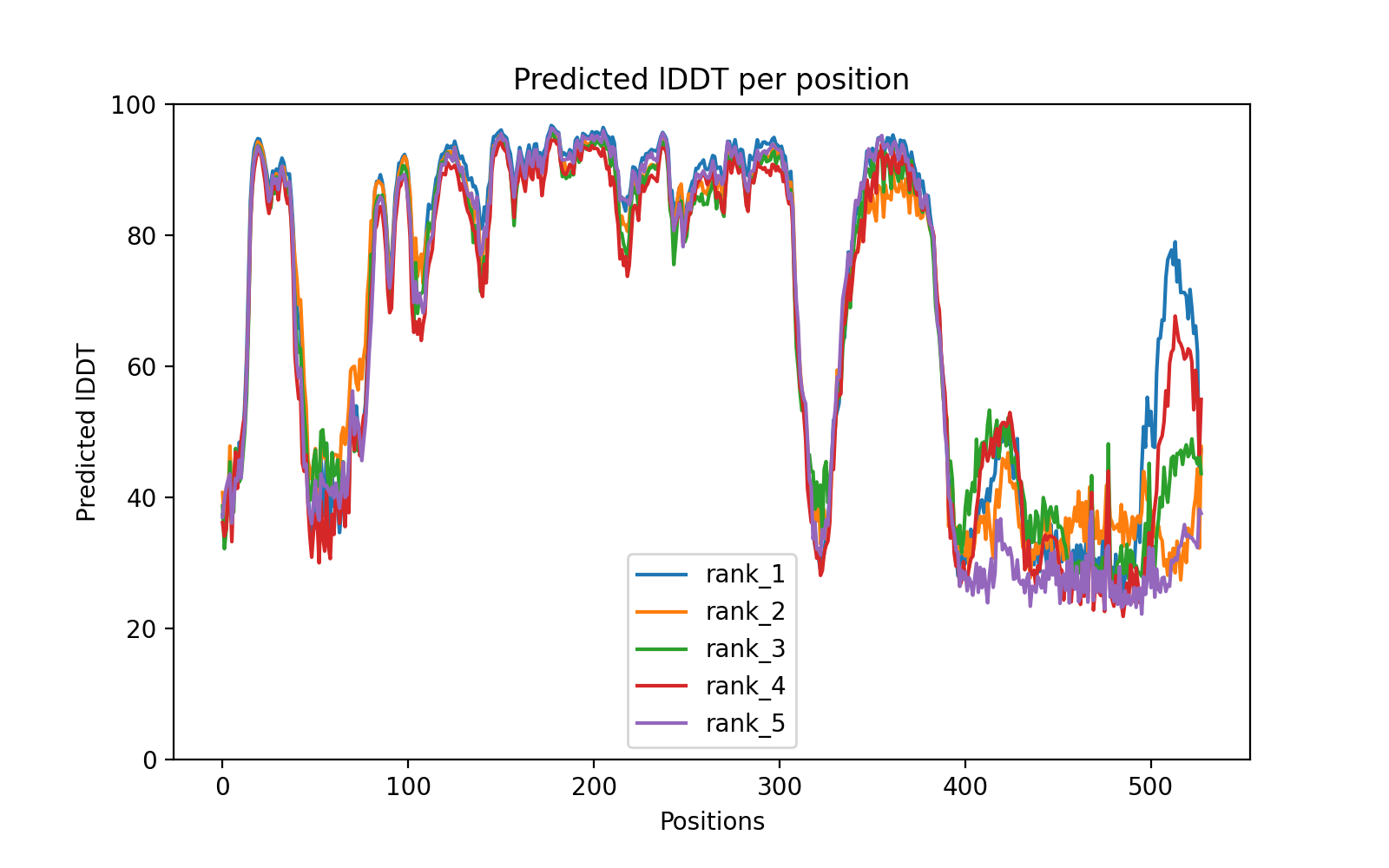

Supplement: Supplementary file 1 [file DataSheet3.zip › Delic_Shuman_Supp_File6/AncGroup3_2f3f8.result/AncGroup3_2f3f8_plddt.png]

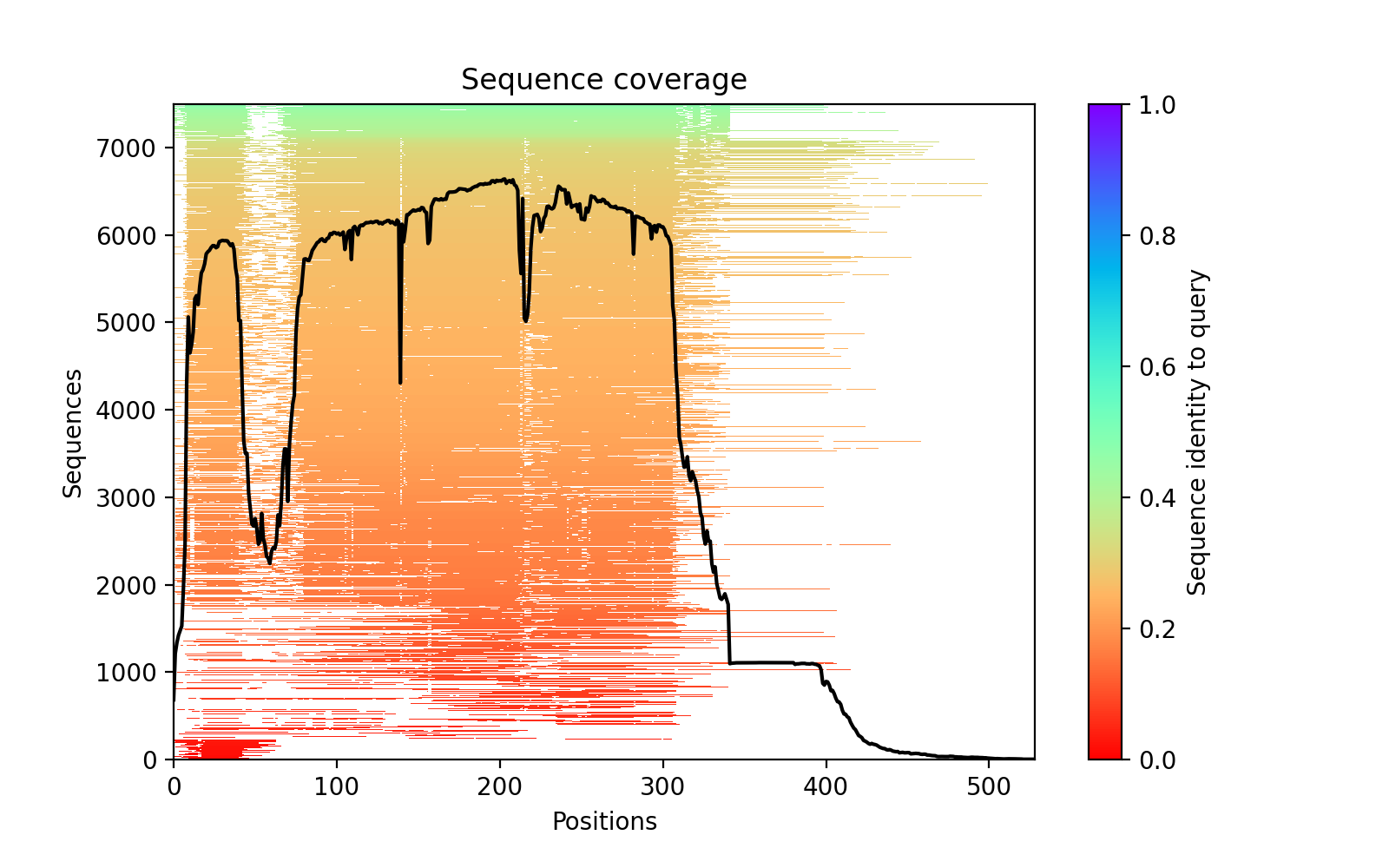

Supplement: Supplementary file 1 [file DataSheet3.zip › Delic_Shuman_Supp_File6/AncGroup3_2f3f8.result/AncGroup3_2f3f8_coverage.png]

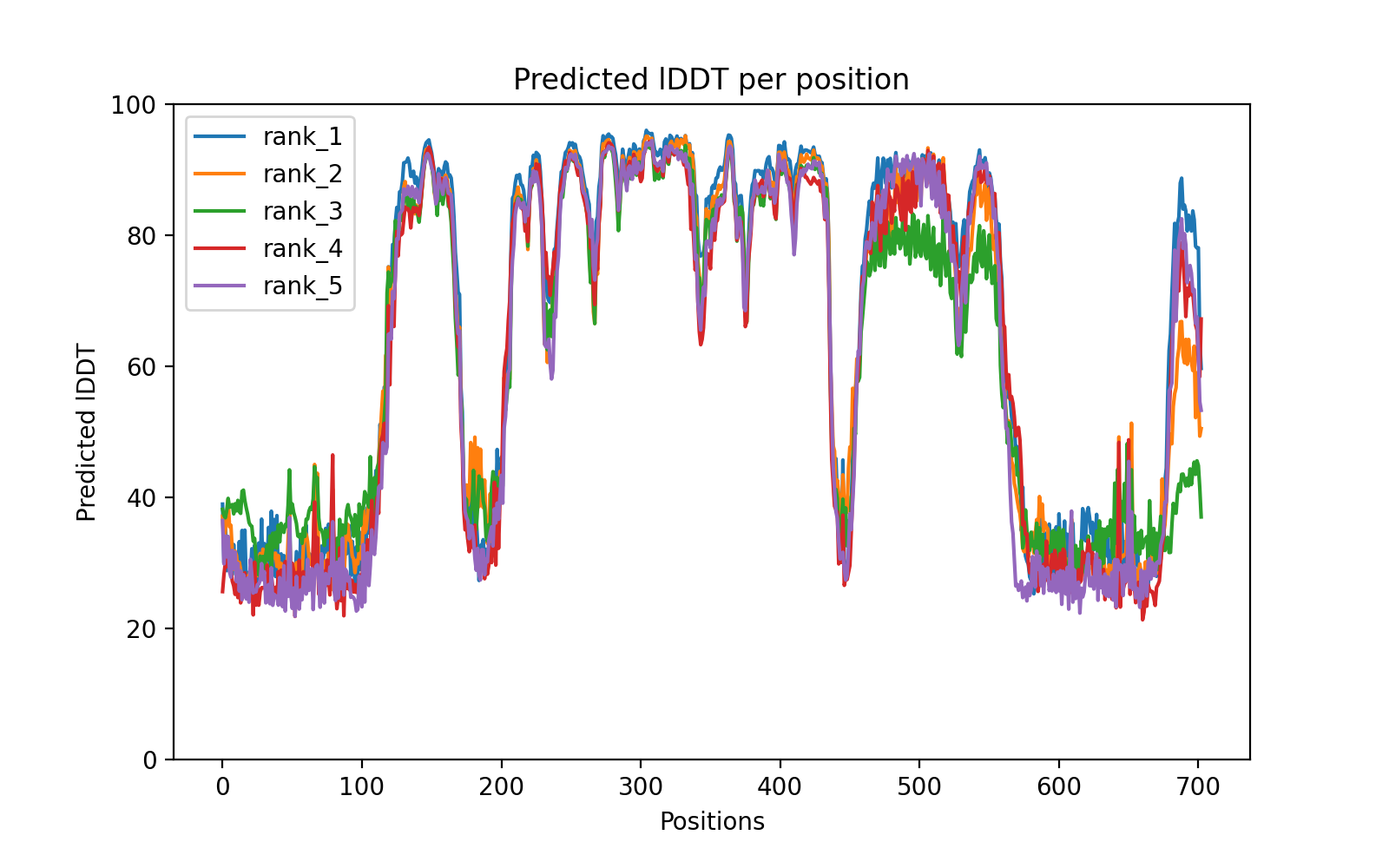

Supplement: Supplementary file 1 [file DataSheet3.zip › Delic_Shuman_Supp_File6/AncGroup2_3fced.result/AncGroup2_3fced_plddt.png]

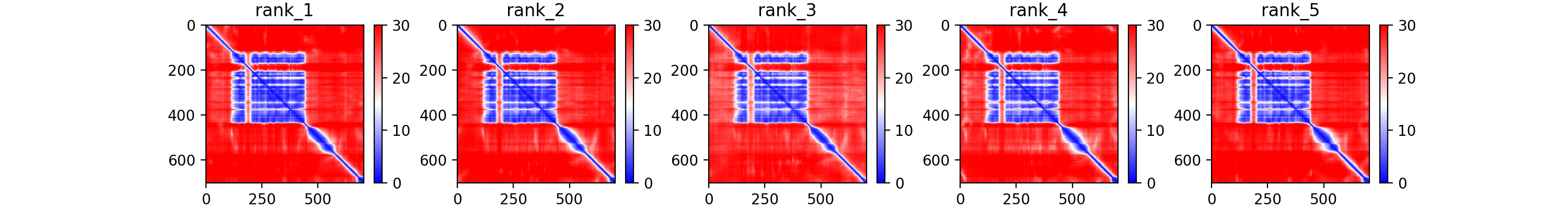

Supplement: Supplementary file 1 [file DataSheet3.zip › Delic_Shuman_Supp_File6/AncGroup2_3fced.result/AncGroup2_3fced_PAE.png]

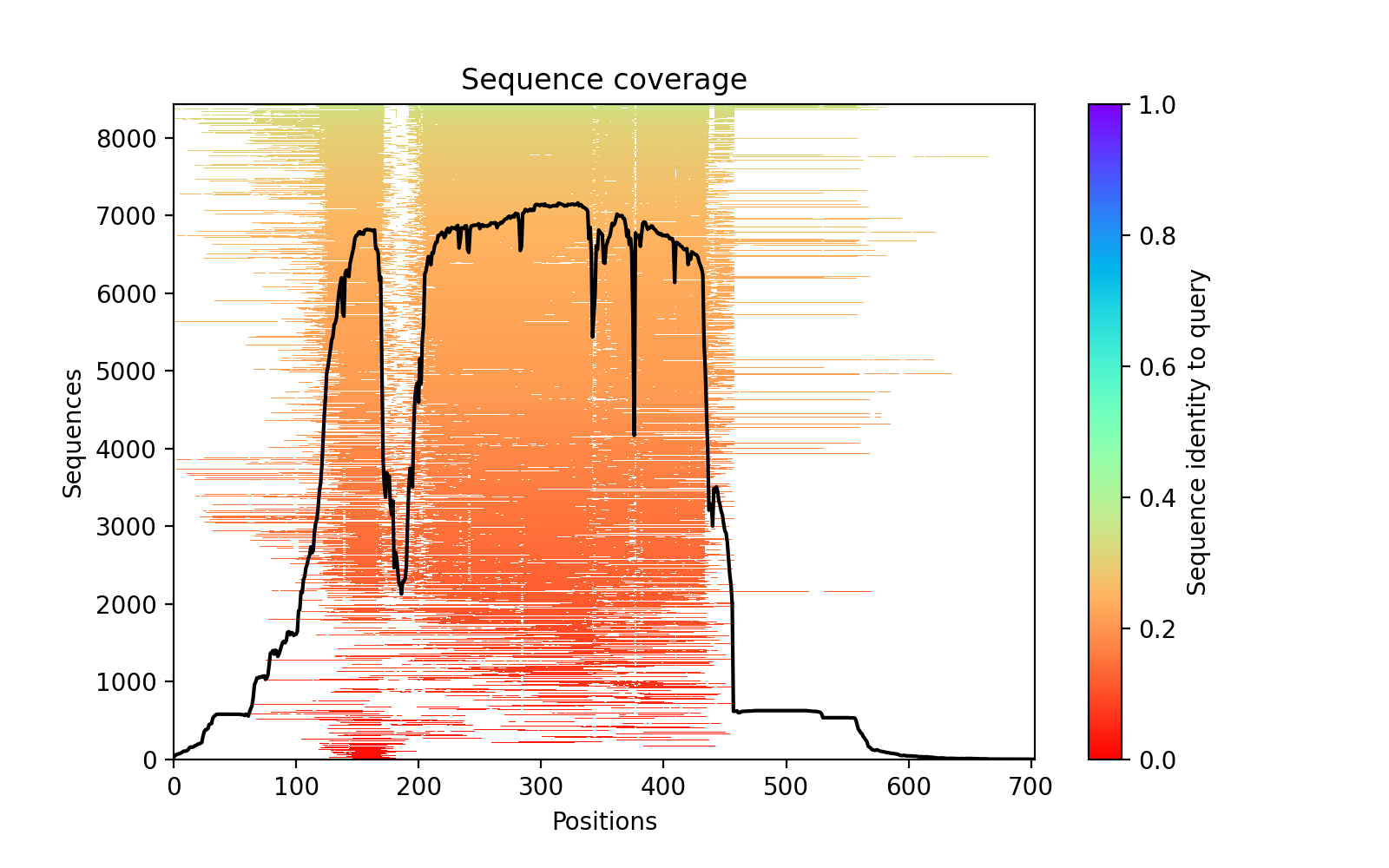

Supplement: Supplementary file 1 [file DataSheet3.zip › Delic_Shuman_Supp_File6/AncGroup2_3fced.result/AncGroup2_3fced_coverage.png]

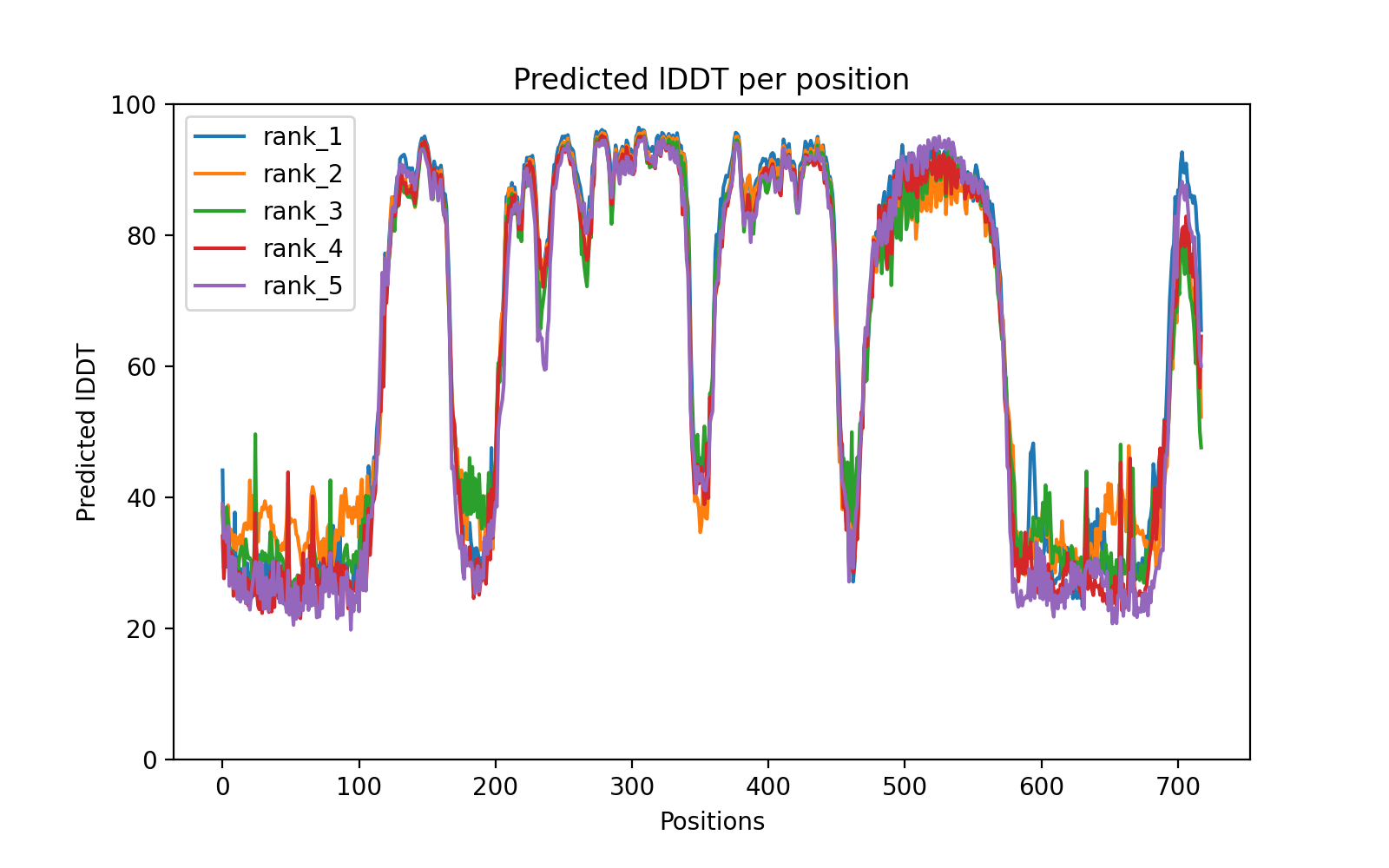

Supplement: Supplementary file 1 [file DataSheet3.zip › Delic_Shuman_Supp_File6/AncGroup1_1a383.result/AncGroup1_1a383_plddt.png]

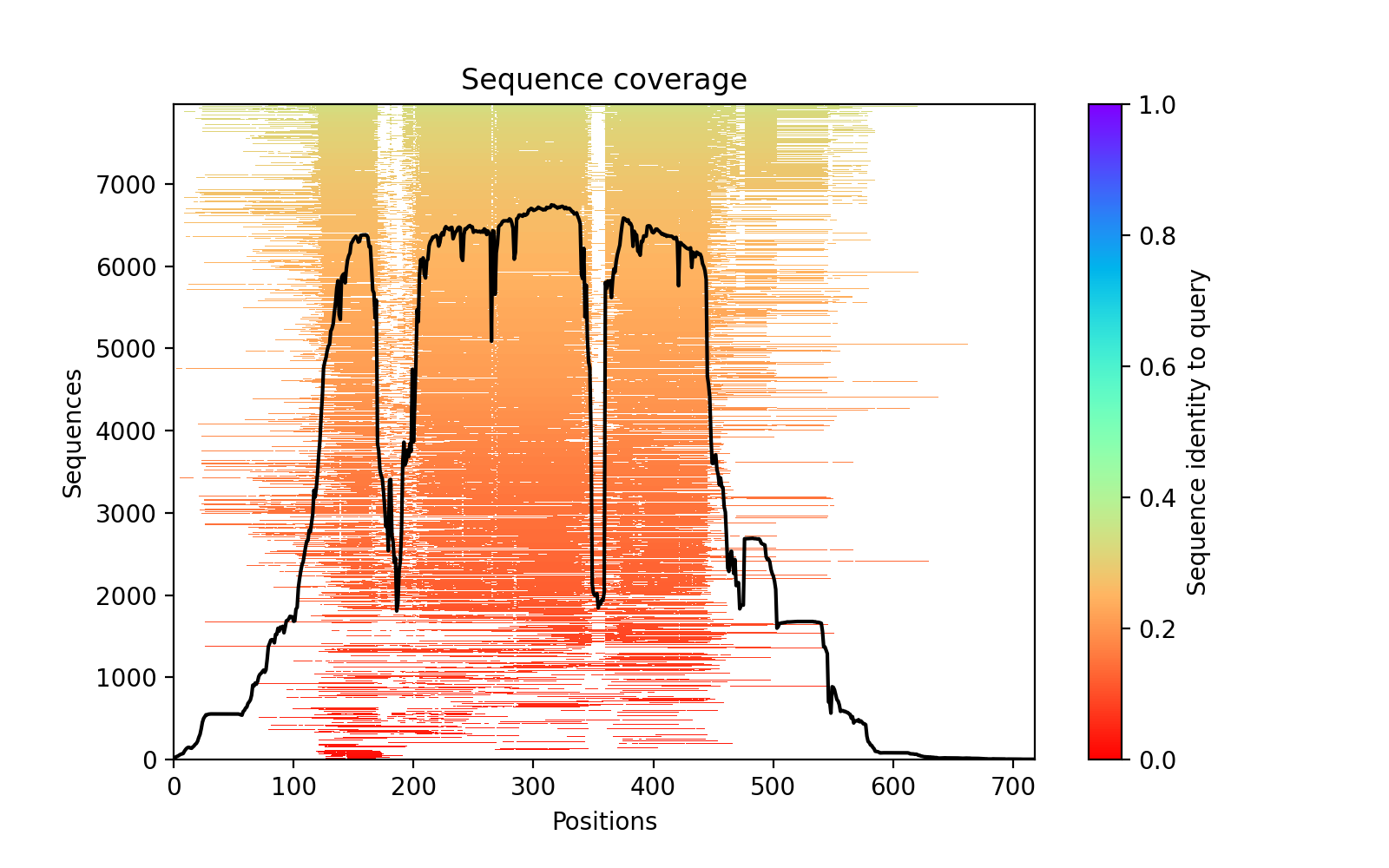

Supplement: Supplementary file 1 [file DataSheet3.zip › Delic_Shuman_Supp_File6/AncGroup1_1a383.result/AncGroup1_1a383_coverage.png]

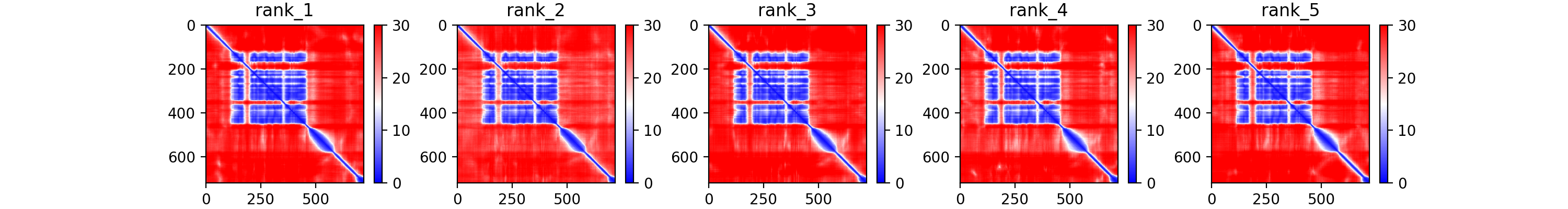

Supplement: Supplementary file 1 [file DataSheet3.zip › Delic_Shuman_Supp_File6/AncGroup1_1a383.result/AncGroup1_1a383_PAE.png]

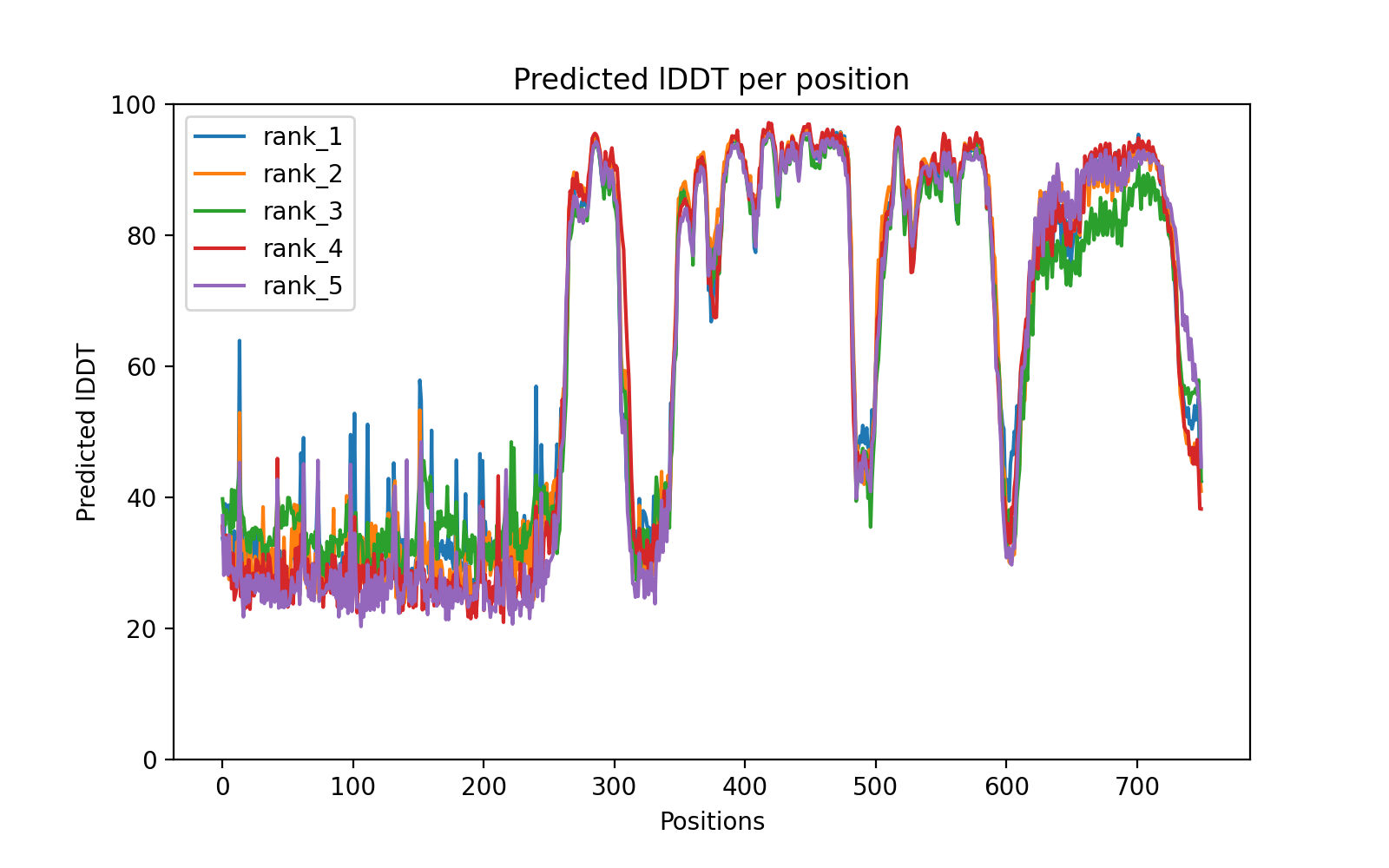

Supplement: Supplementary file 1 [file DataSheet3.zip › Delic_Shuman_Supp_File6/AncGroup4_1_750_91333.result/AncGroup4_1_750_91333_plddt.png]

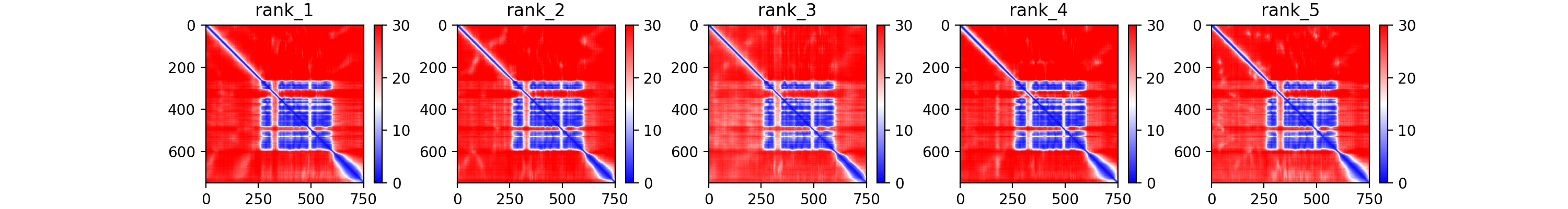

Supplement: Supplementary file 1 [file DataSheet3.zip › Delic_Shuman_Supp_File6/AncGroup4_1_750_91333.result/AncGroup4_1_750_91333_PAE.png]

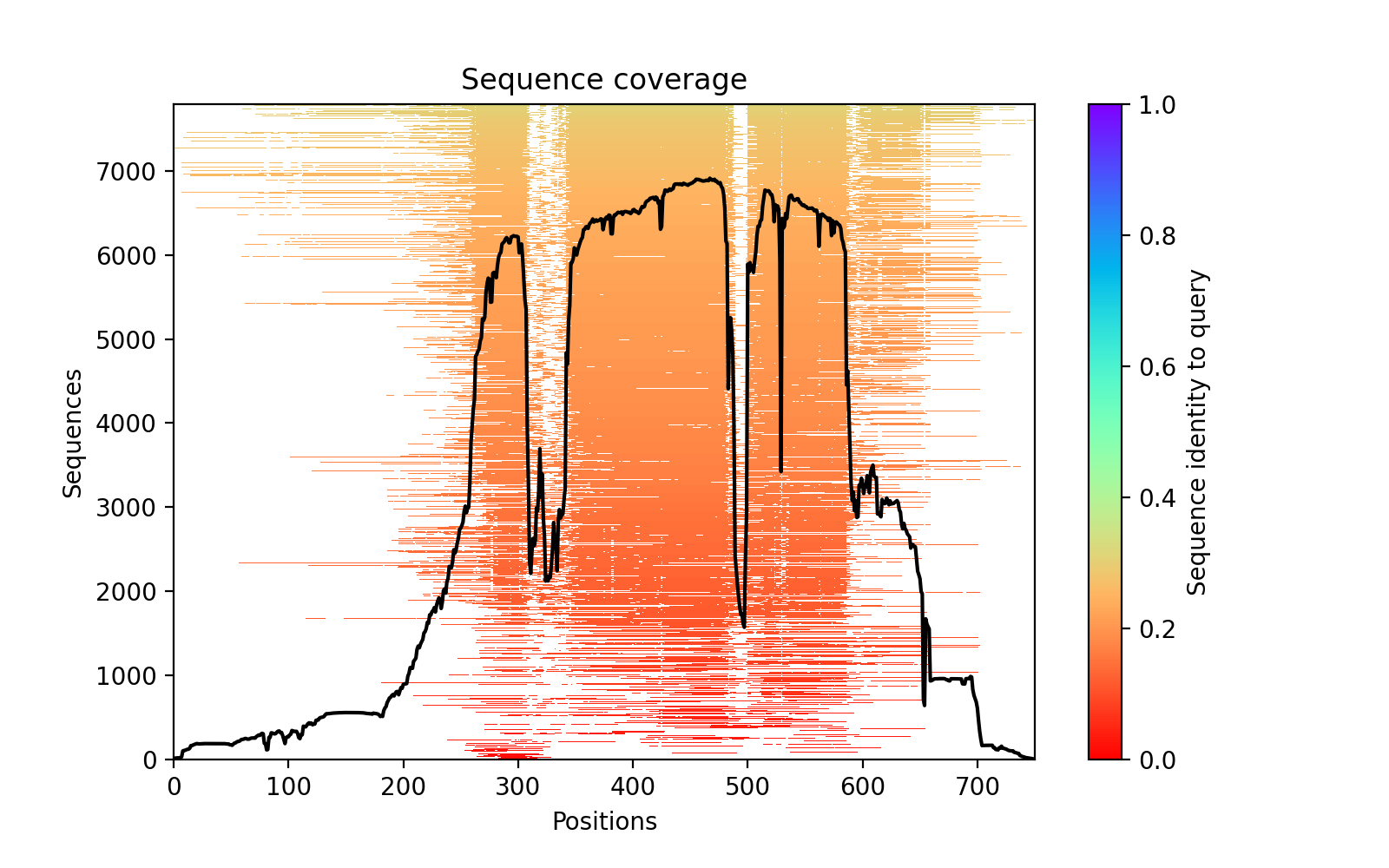

Supplement: Supplementary file 1 [file DataSheet3.zip › Delic_Shuman_Supp_File6/AncGroup4_1_750_91333.result/AncGroup4_1_750_91333_coverage.png]

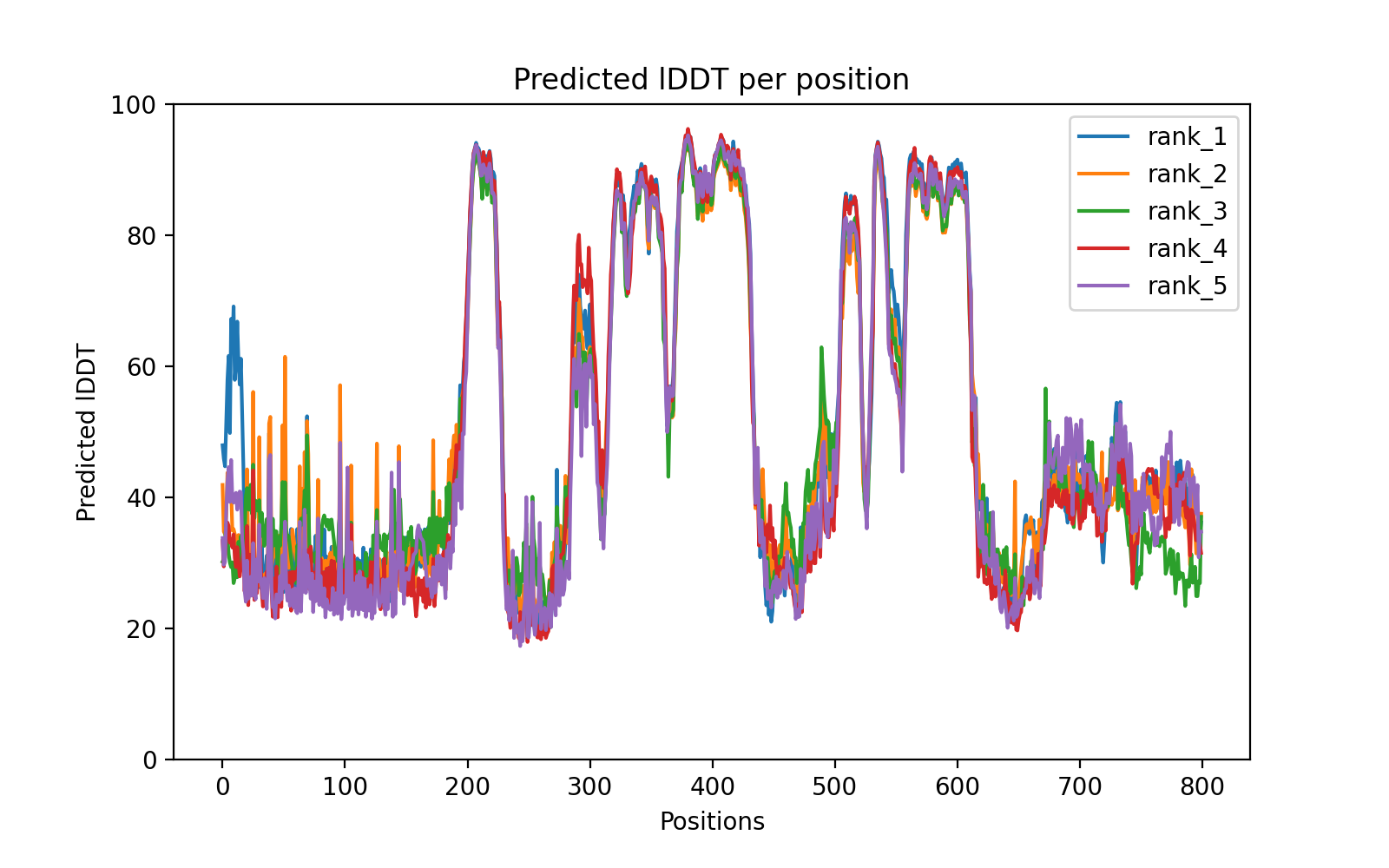

Supplement: Supplementary file 1 [file DataSheet3.zip › Delic_Shuman_Supp_File6/AncGroup6B_450_1250_60852.result/AncChlorophyte_450_1250_60852_plddt.png]

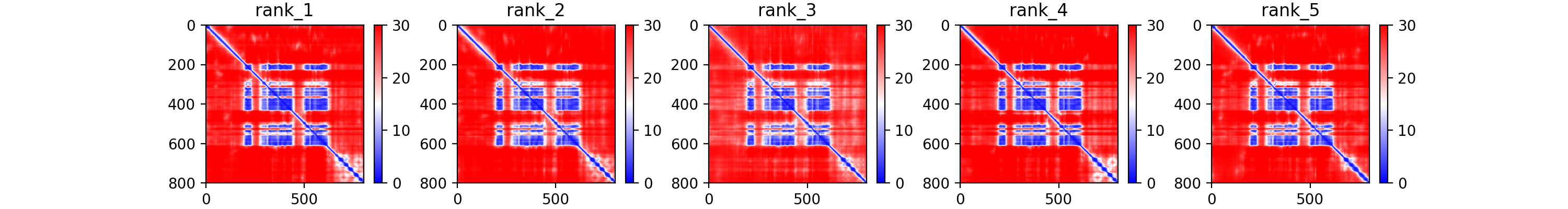

Supplement: Supplementary file 1 [file DataSheet3.zip › Delic_Shuman_Supp_File6/AncGroup6B_450_1250_60852.result/AncChlorophyte_450_1250_60852_PAE.png]

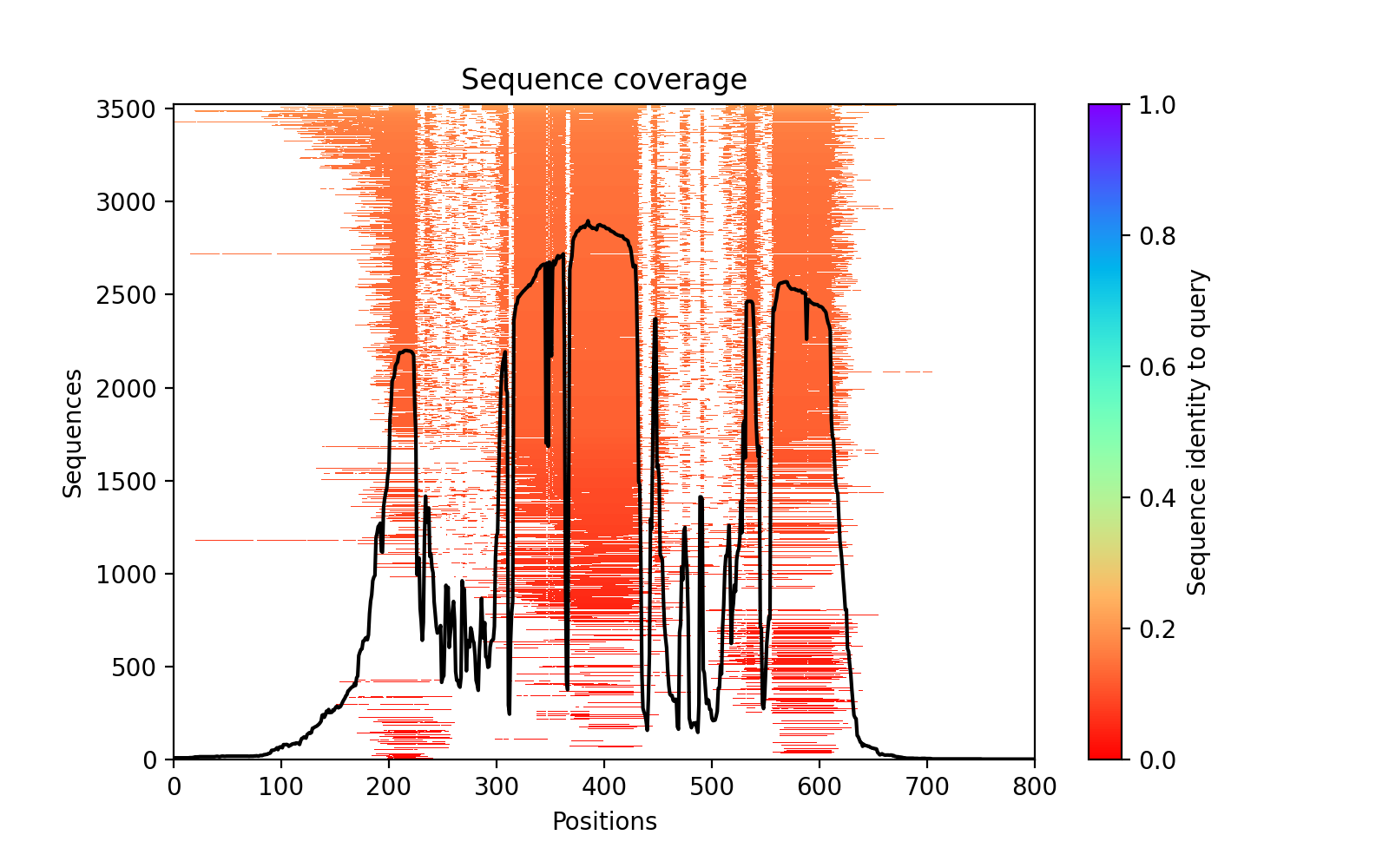

Supplement: Supplementary file 1 [file DataSheet3.zip › Delic_Shuman_Supp_File6/AncGroup6B_450_1250_60852.result/AncChlorophyte_450_1250_60852_coverage.png]

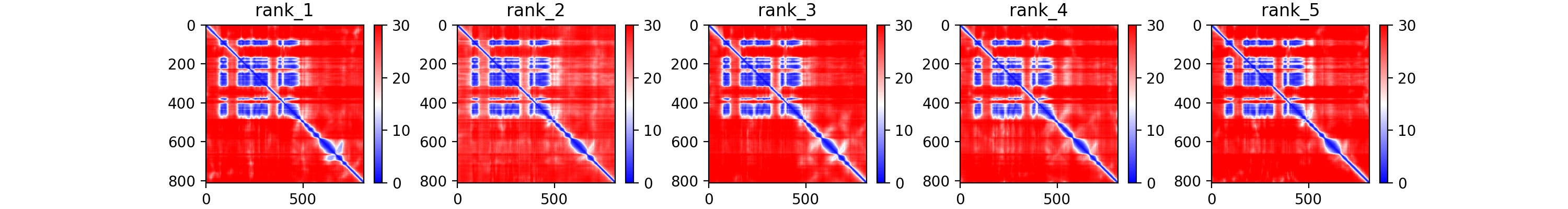

Supplement: Supplementary file 1 [file DataSheet3.zip › Delic_Shuman_Supp_File6/AncGroup7_450___47750.result/AncProt_450___47750_PAE.png]

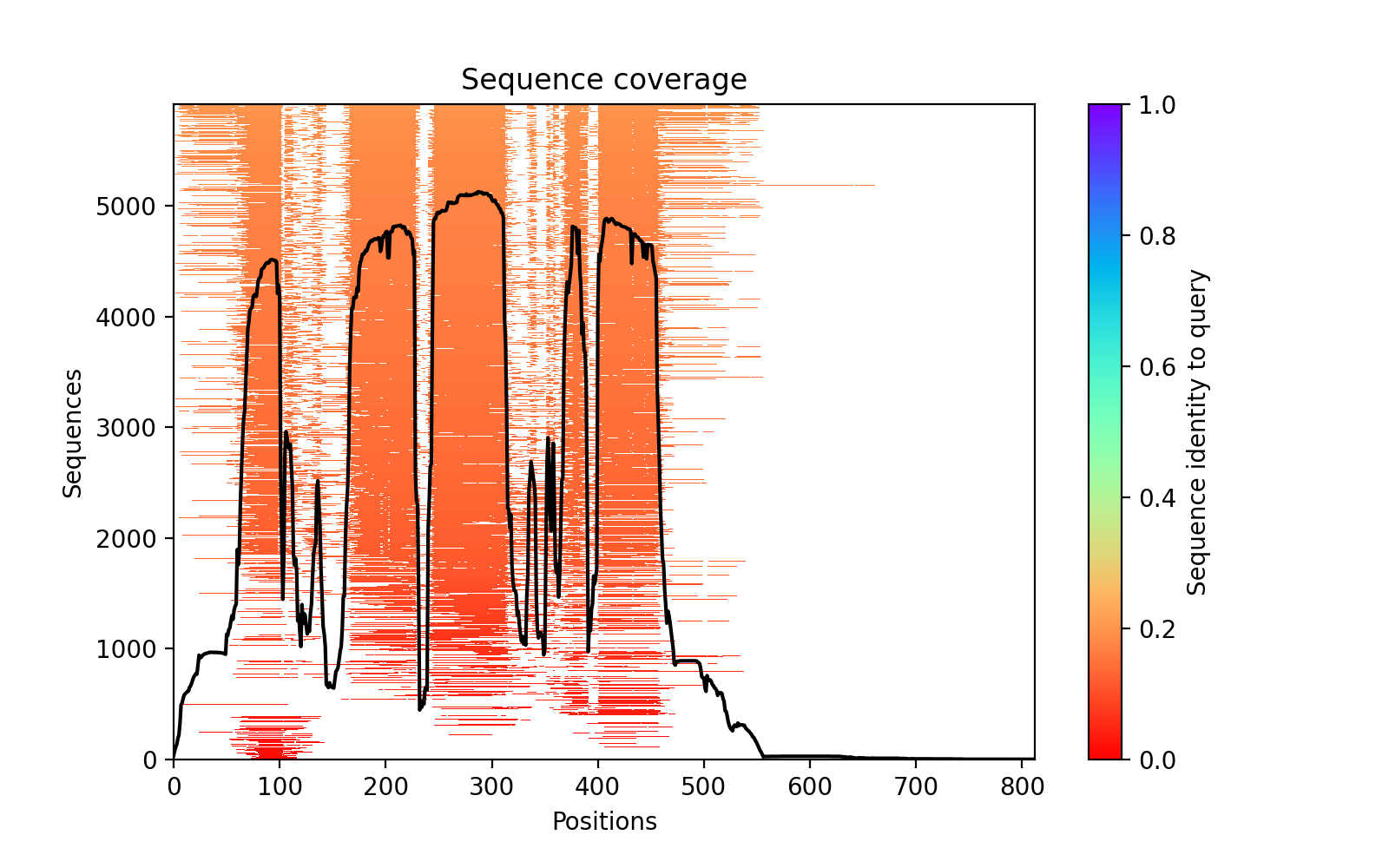

Supplement: Supplementary file 1 [file DataSheet3.zip › Delic_Shuman_Supp_File6/AncGroup7_450___47750.result/AncProt_450___47750_coverage.png]

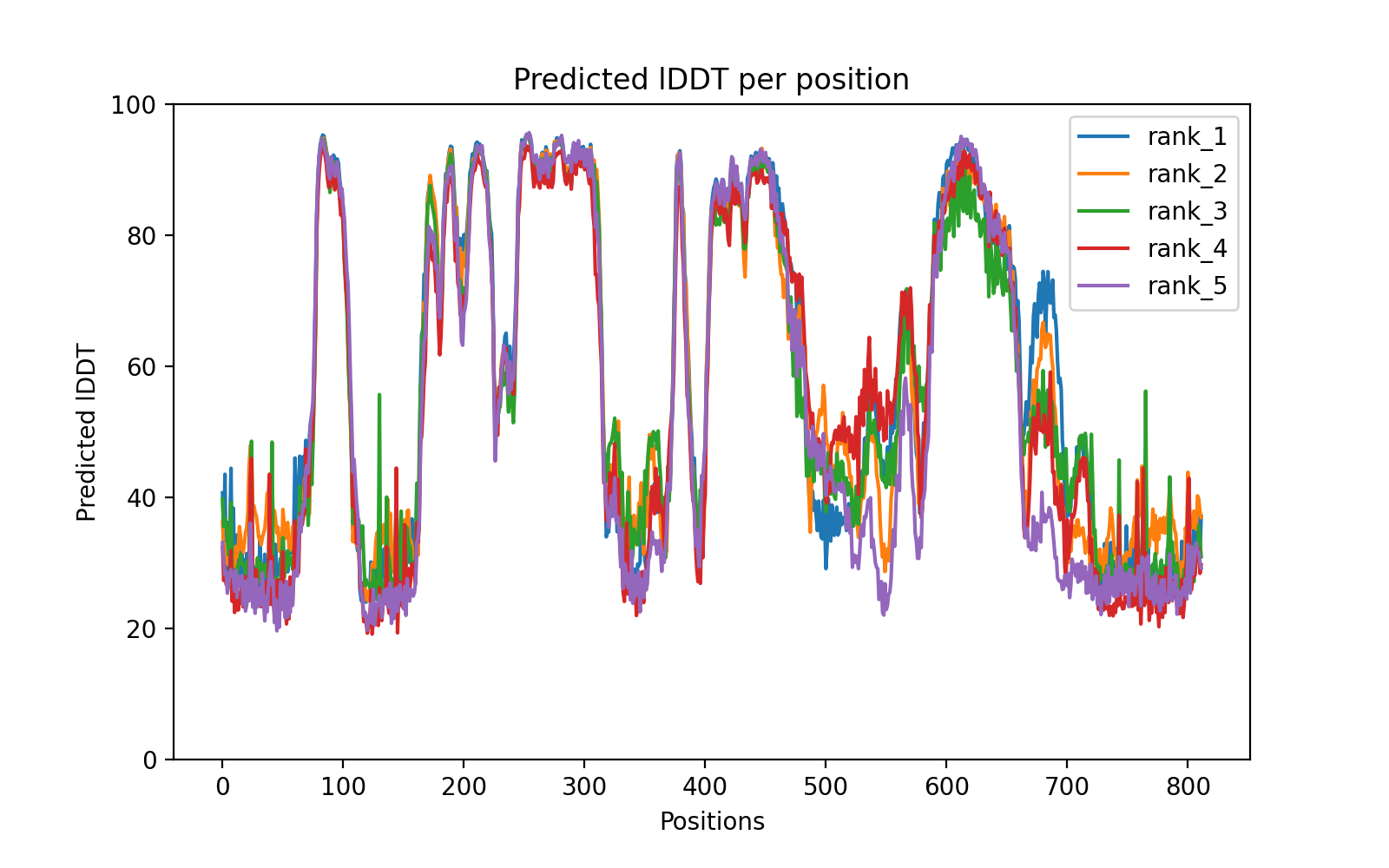

Supplement: Supplementary file 1 [file DataSheet3.zip › Delic_Shuman_Supp_File6/AncGroup7_450___47750.result/AncProt_450___47750_plddt.png]

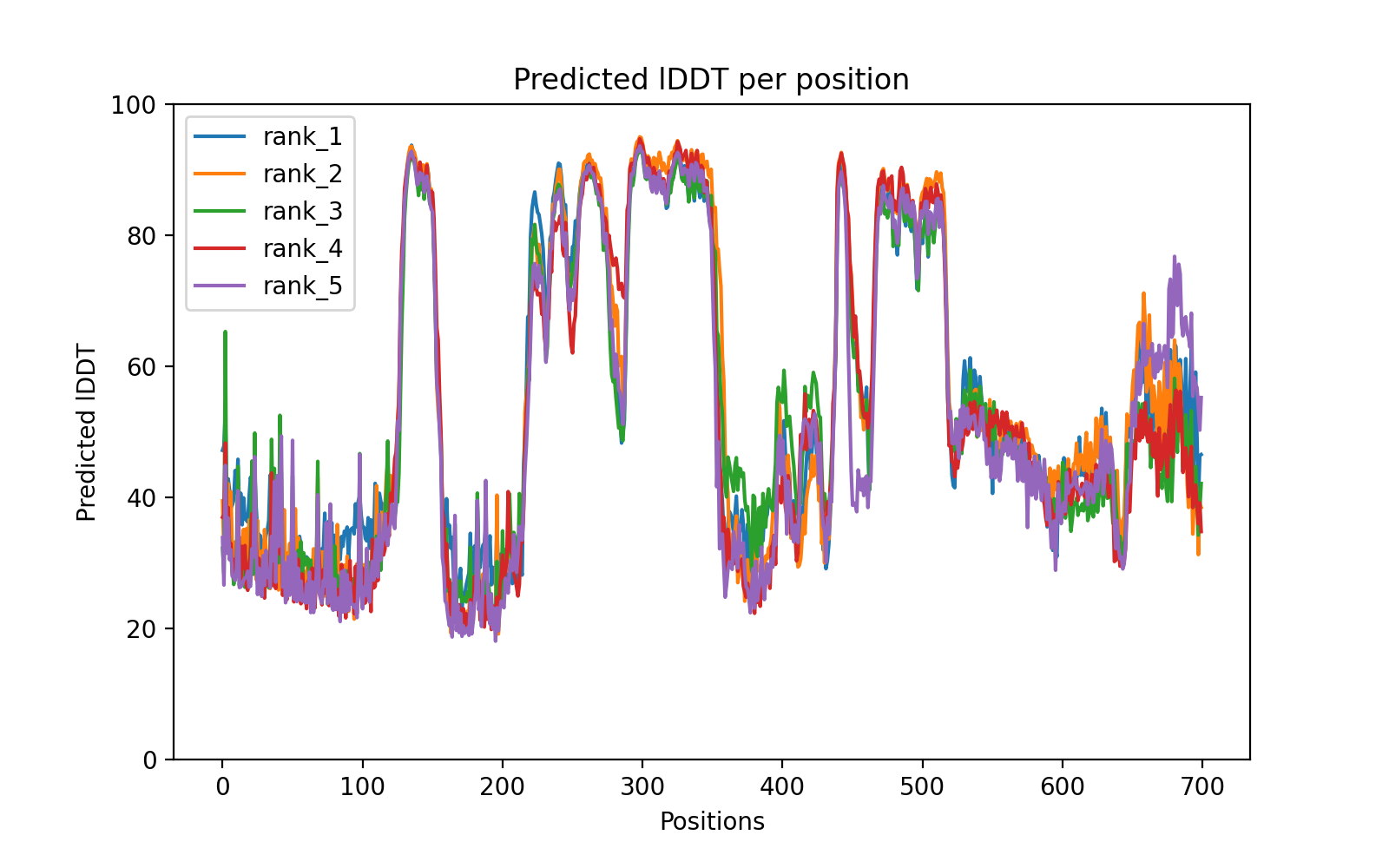

Supplement: Supplementary file 1 [file DataSheet3.zip › Delic_Shuman_Supp_File6/AncGroup6_450_1150_73d48.result/AncGeneralChlorophyte_450_1150_73d48_plddt.png]

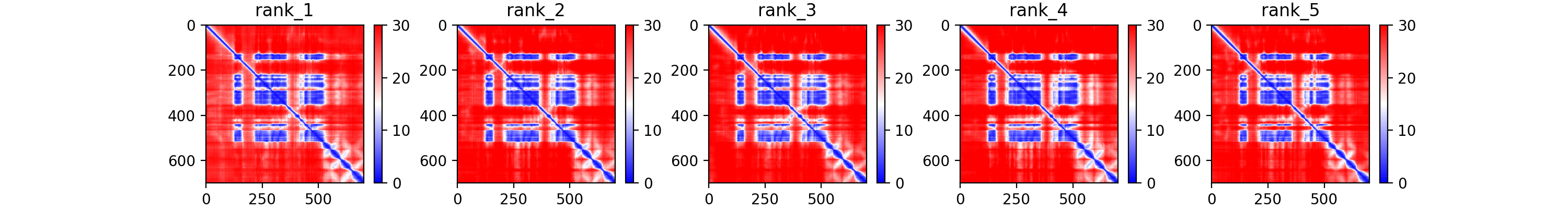

Supplement: Supplementary file 1 [file DataSheet3.zip › Delic_Shuman_Supp_File6/AncGroup6_450_1150_73d48.result/AncGeneralChlorophyte_450_1150_73d48_PAE.png]

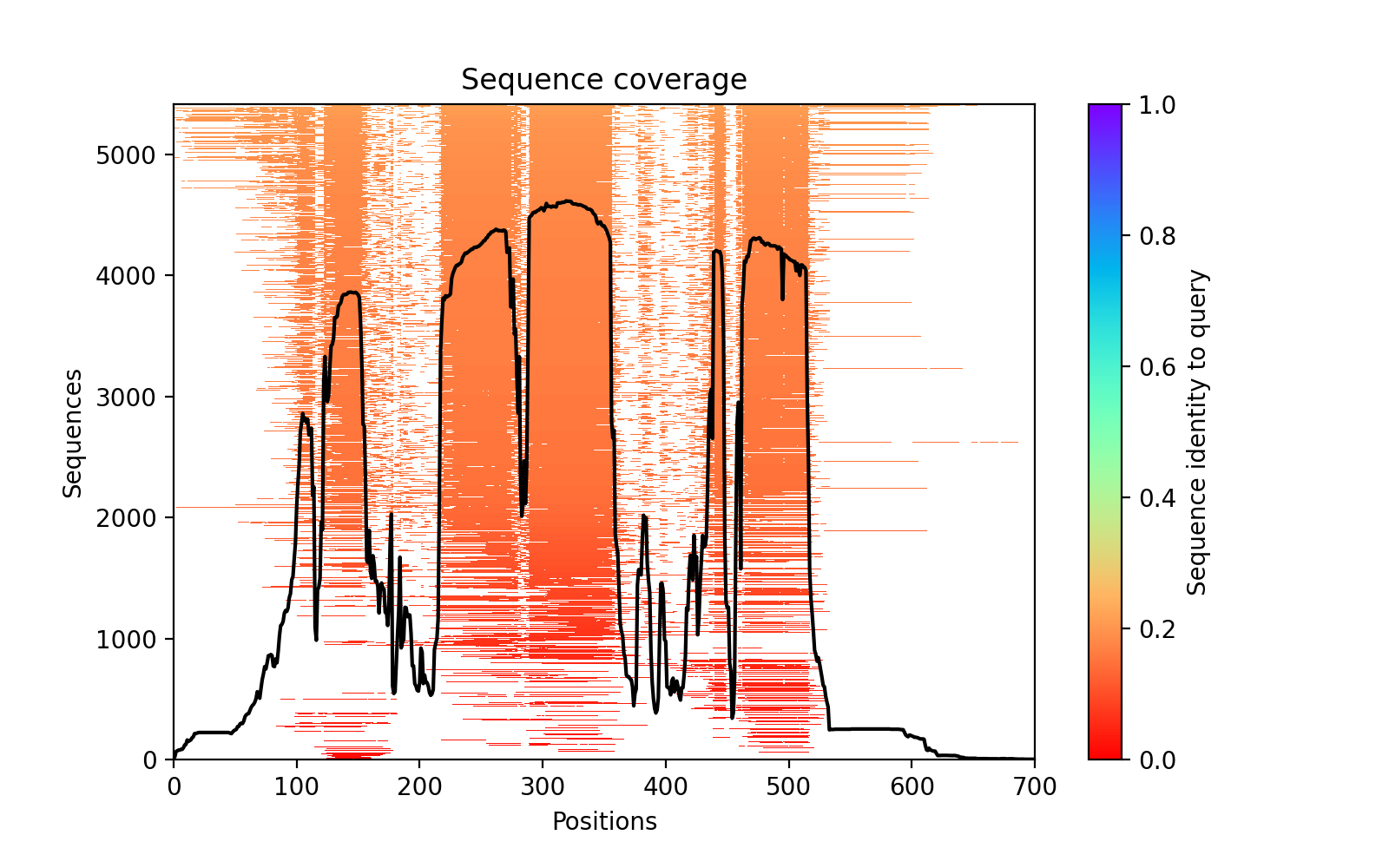

Supplement: Supplementary file 1 [file DataSheet3.zip › Delic_Shuman_Supp_File6/AncGroup6_450_1150_73d48.result/AncGeneralChlorophyte_450_1150_73d48_coverage.png]

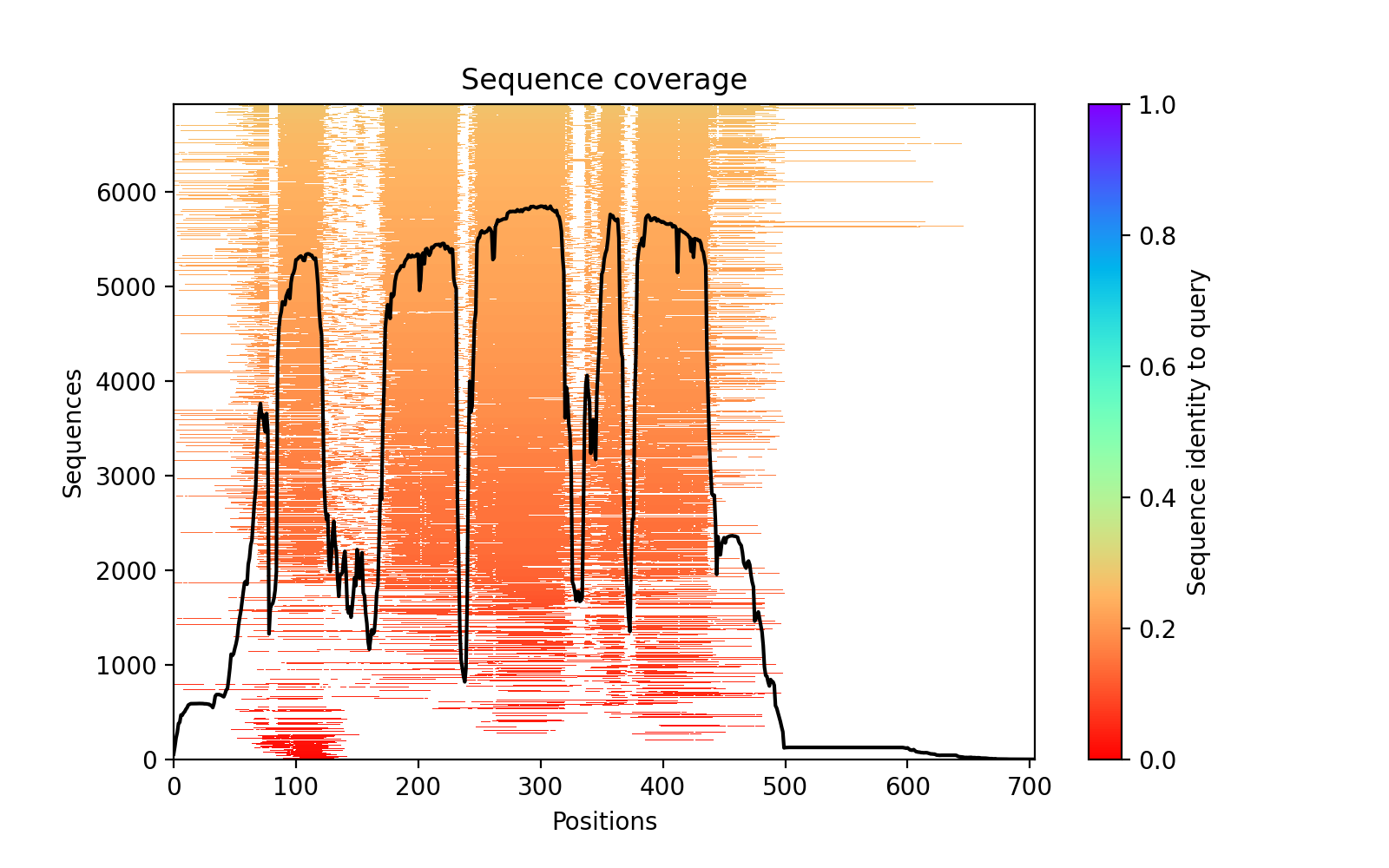

Supplement: Supplementary file 1 [file DataSheet3.zip › Delic_Shuman_Supp_File6/AncOpis_200_900_b2ee0.result/AncOpis_200_900_b2ee0_coverage.png]

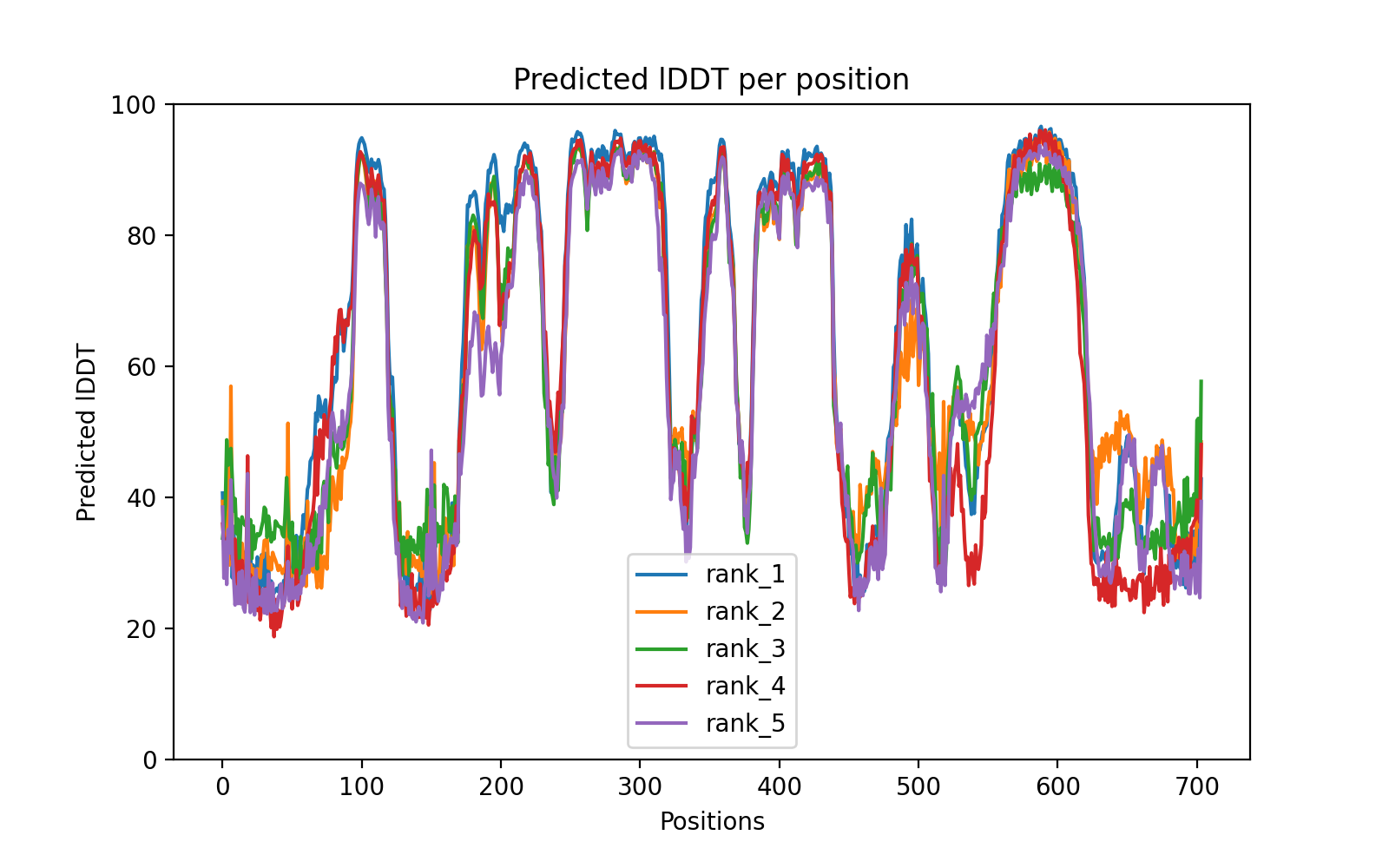

Supplement: Supplementary file 1 [file DataSheet3.zip › Delic_Shuman_Supp_File6/AncOpis_200_900_b2ee0.result/AncOpis_200_900_b2ee0_plddt.png]

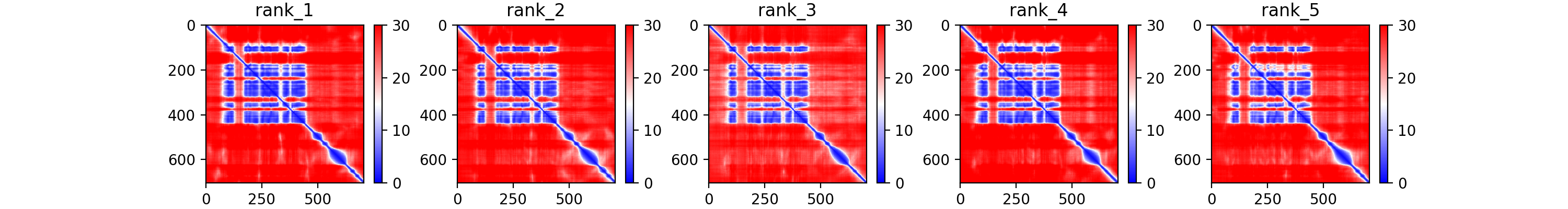

Supplement: Supplementary file 1 [file DataSheet3.zip › Delic_Shuman_Supp_File6/AncOpis_200_900_b2ee0.result/AncOpis_200_900_b2ee0_PAE.png]

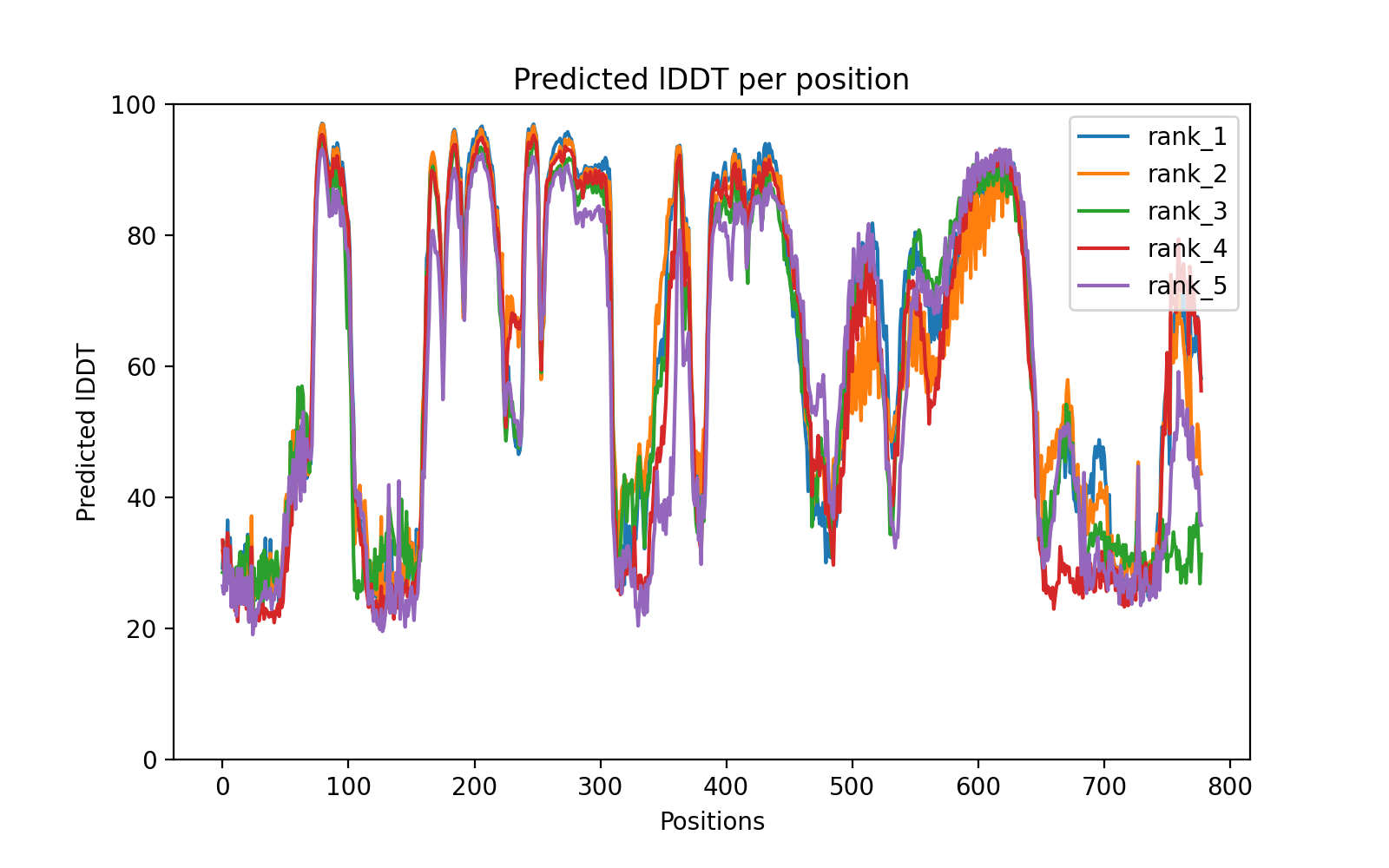

Supplement: Supplementary file 1 [file DataSheet3.zip › Delic_Shuman_Supp_File6/AncGroup8_200_978_9bb52.result/AncCiliates_200_978_9bb52_plddt.png]

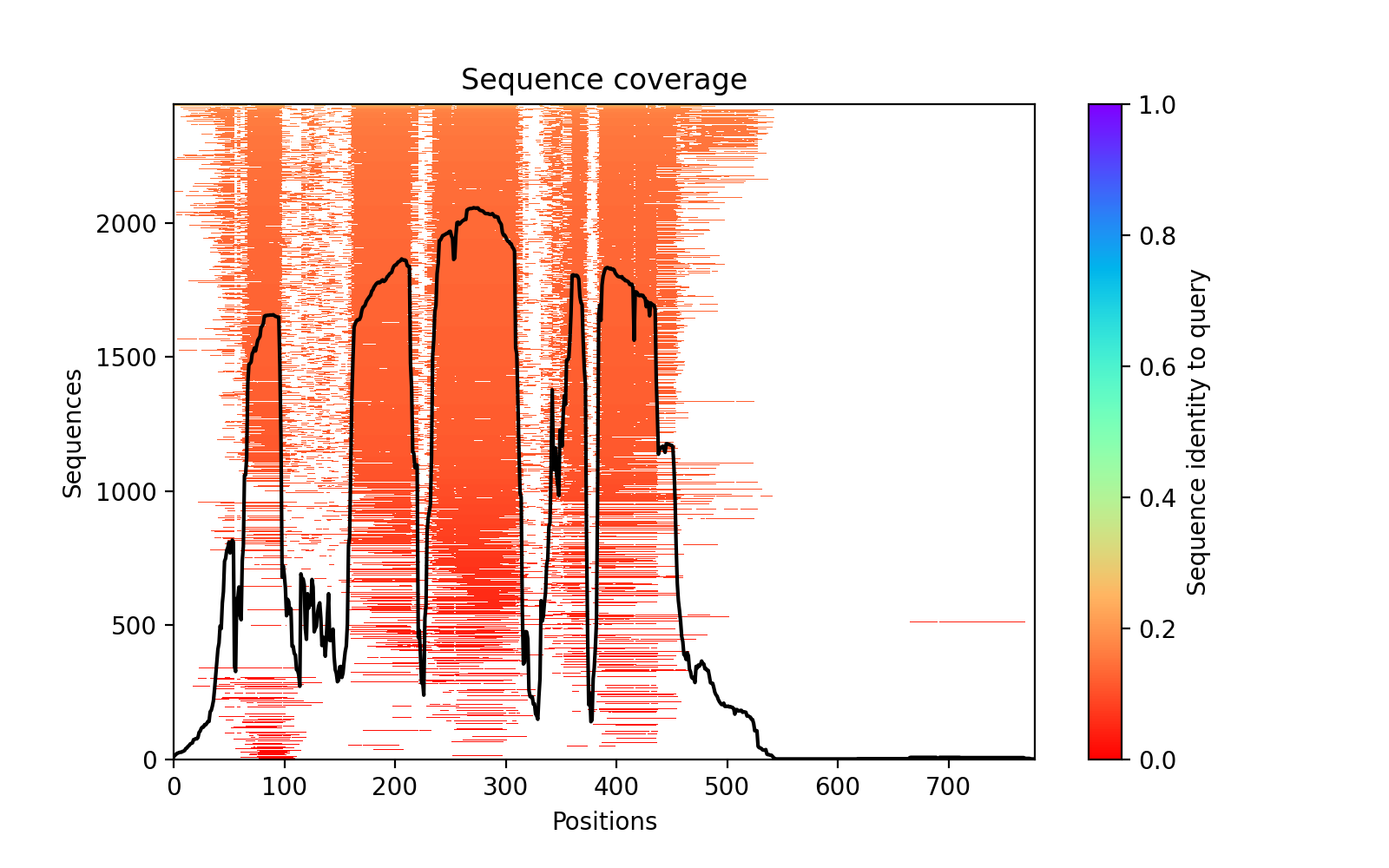

Supplement: Supplementary file 1 [file DataSheet3.zip › Delic_Shuman_Supp_File6/AncGroup8_200_978_9bb52.result/AncCiliates_200_978_9bb52_coverage.png]

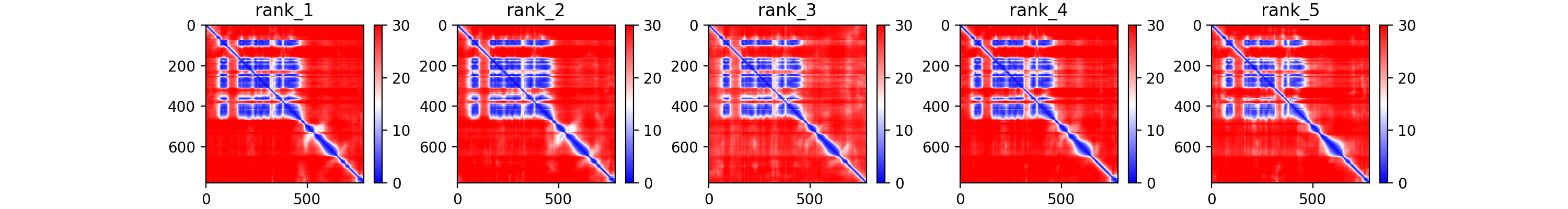

Supplement: Supplementary file 1 [file DataSheet3.zip › Delic_Shuman_Supp_File6/AncGroup8_200_978_9bb52.result/AncCiliates_200_978_9bb52_PAE.png]

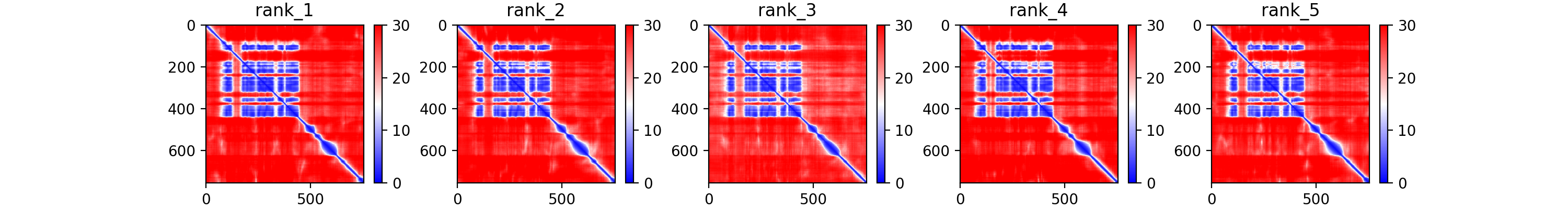

Supplement: Supplementary file 1 [file DataSheet3.zip › Delic_Shuman_Supp_File6/AncGroup5_200_954_98a30.result/AncGroup5_200_954_98a30_PAE.png]

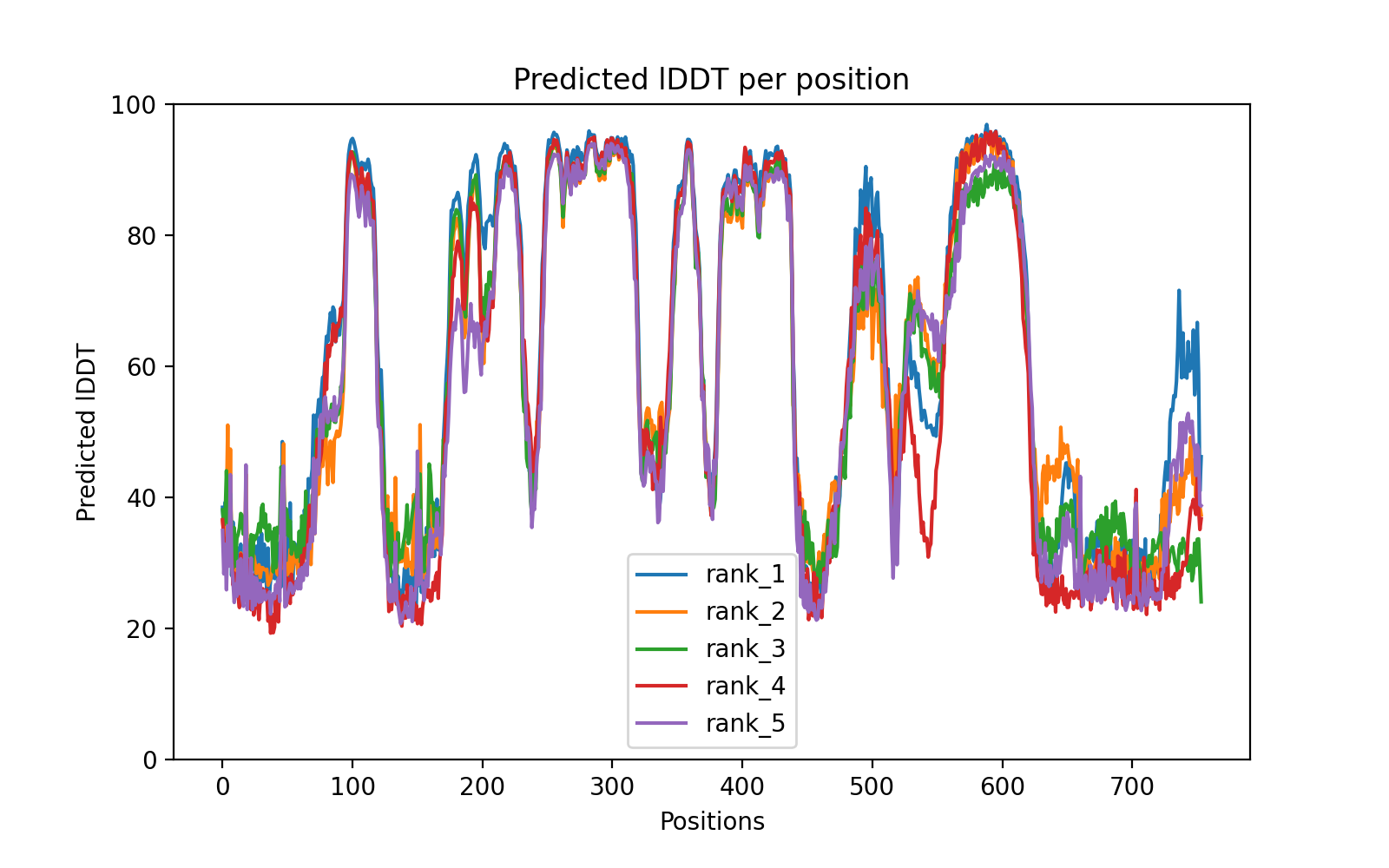

Supplement: Supplementary file 1 [file DataSheet3.zip › Delic_Shuman_Supp_File6/AncGroup5_200_954_98a30.result/AncGroup5_200_954_98a30_plddt.png]

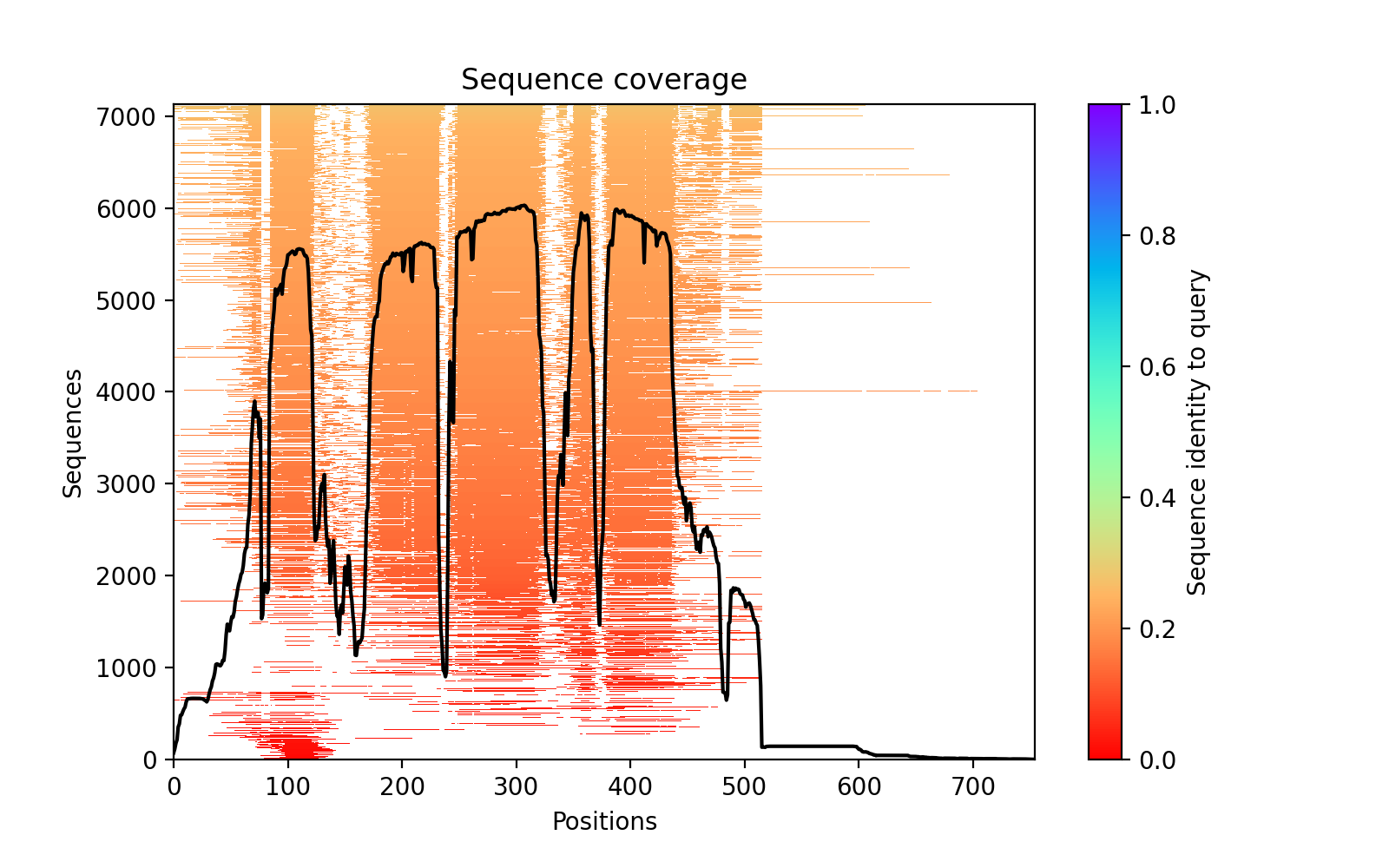

Supplement: Supplementary file 1 [file DataSheet3.zip › Delic_Shuman_Supp_File6/AncGroup5_200_954_98a30.result/AncGroup5_200_954_98a30_coverage.png]

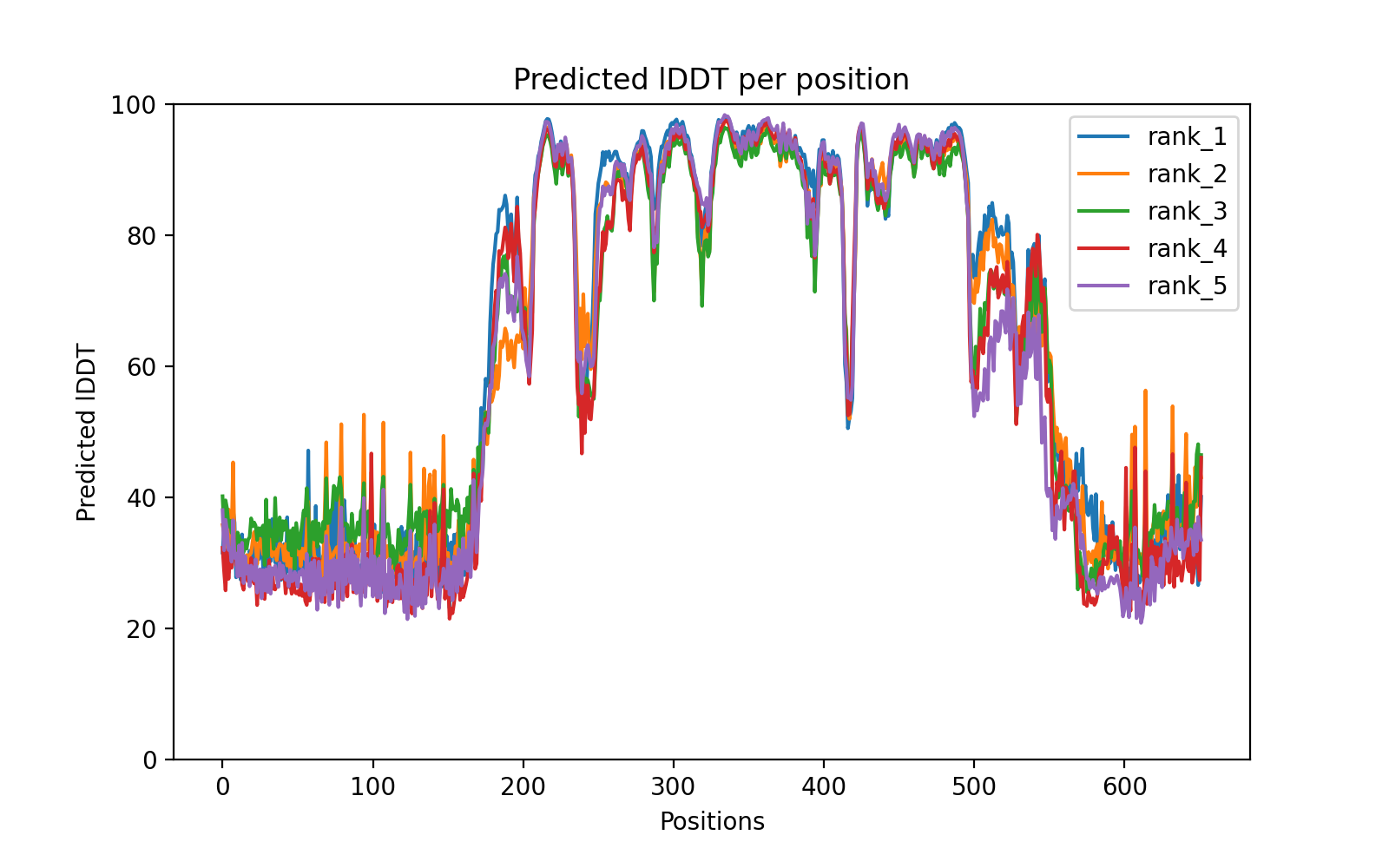

Supplement: Supplementary file 1 [file DataSheet3.zip › Delic_Shuman_Supp_File6/AncGroup6A_2173e.result/AncChlorophyte_Volvo_2173e_plddt.png]

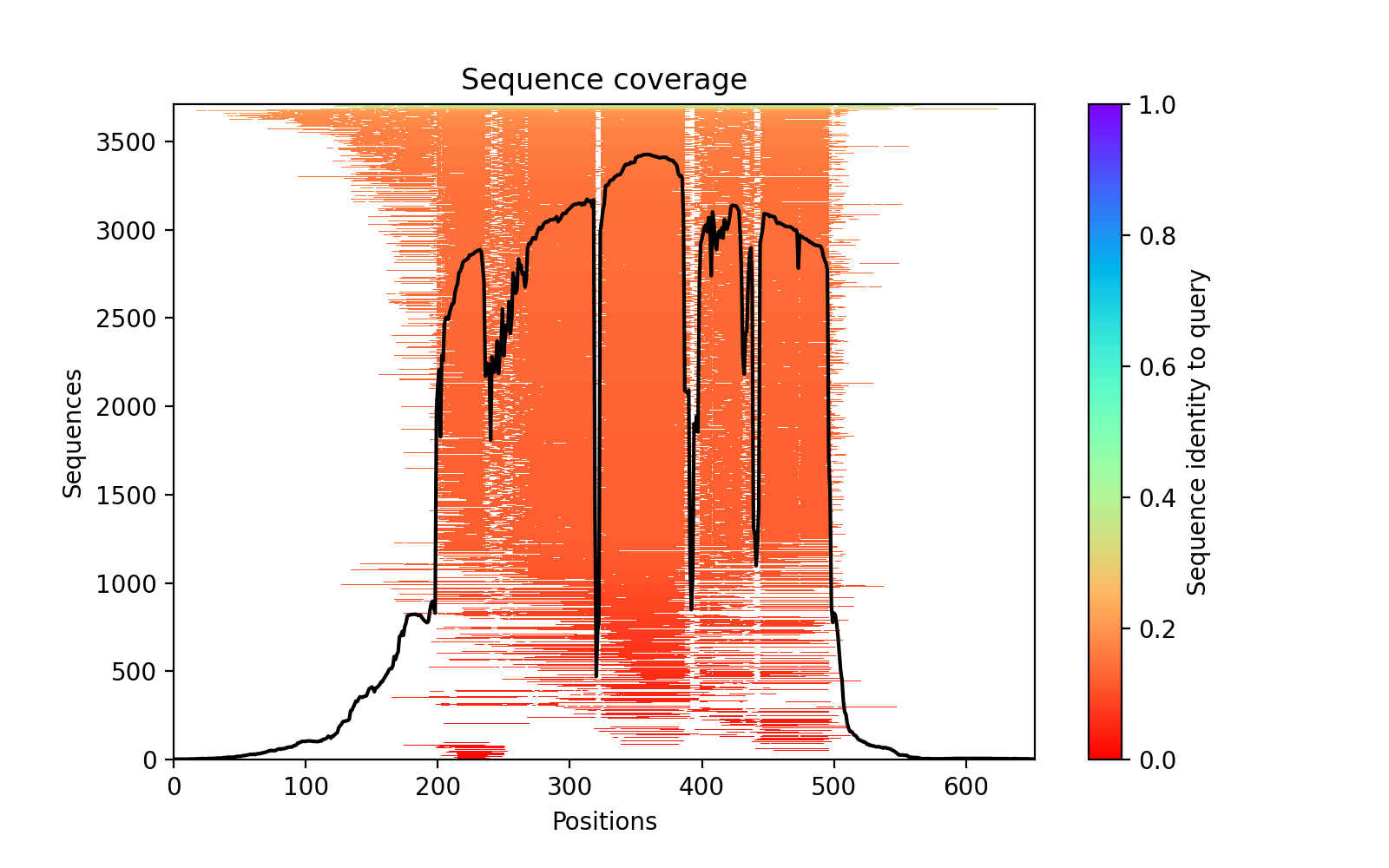

Supplement: Supplementary file 1 [file DataSheet3.zip › Delic_Shuman_Supp_File6/AncGroup6A_2173e.result/AncChlorophyte_Volvo_2173e_coverage.png]

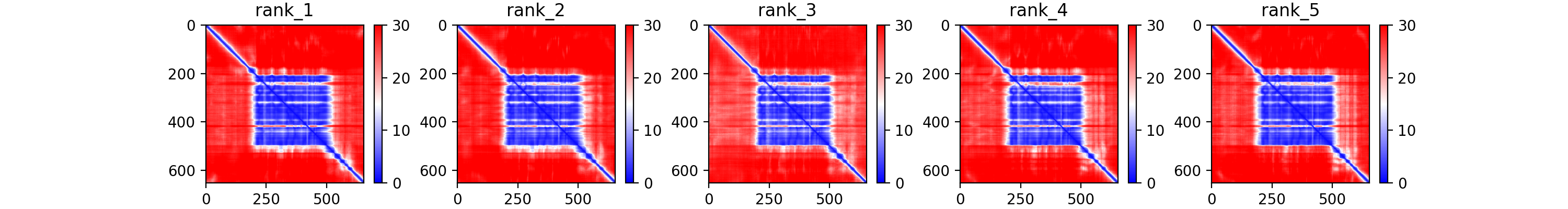

Supplement: Supplementary file 1 [file DataSheet3.zip › Delic_Shuman_Supp_File6/AncGroup6A_2173e.result/AncChlorophyte_Volvo_2173e_PAE.png]
