# Supplementary material for: The evolutionary origins and ancestral features of septins
Source: Front Cell Dev Biol. 2024 Jun 26;12:1406966. doi: 10.3389/fcell.2024.1406966 (PMC11238149; doi:10.3389/fcell.2024.1406966)
Supplement: Supplementary file 2 [file DataSheet4.zip › SupFile9_20240430.docx]

**XP_662412_1**

**April 29, 2024**

Below is a screenshot of the genomic context of aspE (AN10595) in *A. nidulans*. The gene directly following aspE is the annexin ANXC4 (AN10598). At the bottom of the image, RNAseq data shows these two genes have separate expression patterns. On the next page, one can observe XP_662412_1 is a combination of aspE, ANXC4, and the region between them.

Record on NCBI database indicates that the locus page for **XP_662412** was updated to correct this error while the present work was ongoing. This change does not affect the overall conclusions of the study.

**Figure 1. A screenshot of the FungiDB genome browser JBrowse.**

**
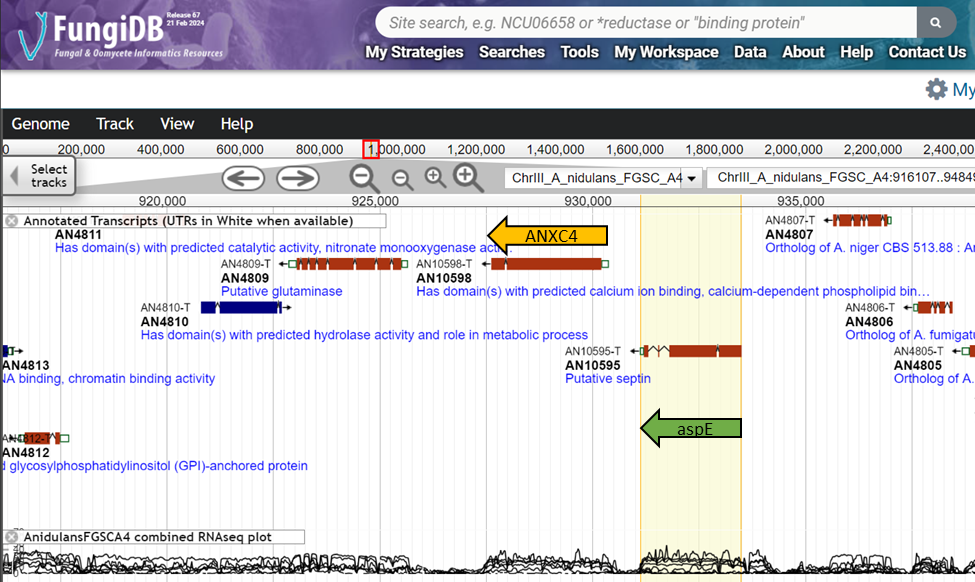
**

**>XP_662412_1_AspE**

MAATATRPRTPGRGQAPIDPPPVPSENGHSQTKDSSRRTSSSLGFLRRSKSTEPIGSKPRGKKMSKAQMEEELRRQREARPKQPPRLPDFSPPPVIETFGGDETRNGVADVTSPLSPSQSRPSRSAMSTPVPPDYSDPYARTESMTHRGRYSYASSYVSTVNNPRRLRRRKDPTPYNILVIGARNSGKTSFLNFLRKSLALPPHKHPSRAPDEVEYDSHNPASEGYTSHYLETEIDGERVGLTLWDSQGLEKNVVDIQLRGVTGFLESKFEETLNEEMKVVRSPGARDTHIHCTFLILDPSRLDENIAAAERAAQGTPRASDSKVLGVLDENFDLQVLRTVIGKTTVVPVISKADTITTAHMAYLRKAVWDSLKKANIDPLEILTLEDQEEYTSSEGEDEEDGETSEAEDAAGETEGGHTKEETEPKAPESPTQRSEGSQQDVGSQAVPLLPFSILSPDKYSLQGDGPIGRKFPWGFADPYNPEHCDFLKLKDSVFSEWRSELREASRVIWYERWRTSRLNRHDAIASPKPRSFGGRTGPDFARRPSGLKHAQNGCHCHIPVSLARPVSFGVPRCQSNWKLSELTALESRQSSDRSSGASLHARSSRTLDDAFLCLGHRIYNMAGKSFVESMSLQVNDPRSRGRSRSPSGRTRDRSTSRDPRLPSPGPGPDPARKSGYLLAETVDEKARTRSRSRGASPLRGYRKTSRYDSDSEHEREREREREARDSYTRLRNDRDYYYHSDSGESRGATKRSSQRYSQPPQRSSAQLDAYSDEDIYSDSDDDLAYGDIPGSLERGYYGYKGNSAATRPPSEKPLMTGALNAGTSPRHSAEAVSGYSRYAPGHPARTGPPTSETQSAWAPVPDCEKPGFVPPTSAGDSMPGAFPTTTSGLPTTQYVSSDPVQNPYVQWNTQPPTSGAPYAAPVSAASHQRNPSGDPNLYANPPAFKYAQIDPNVRYSAKPATATTYAPPSKASGQTSDGQYAGVRYTTAPQYSTTATSGSQYVEIAPGSRHTRPASLSVSTNNLSVSGPDPNNPPASPLLEAYKGTYQSISPMPSPILIAPRDDDVSDLEPLDHSTDSERRRRRKSKKSKDEEGGLKEPKSDRSKRGSSRIRHGRHESRDSRGGGPDSVALVSPSTDRRKEVSFYDATDDALALRDALSHSRNIDTKTLIQVLPHLTNHEMLDLRKEYKKHVKIHGKGVNLAKHIRVKLGNSAFGKVCYATALGRWESEAFWANCYYQSGSSRRELLIESLFGRSNGEMREIKESFKDSRYLDSLEKCMKAELKADKFRTAVLLALEEGRQSERDPIDAELVHRDVQALHAALVSRNGGETAMIYIIVRRSDSHLREVLRAYDKIYQRNFARDMIQKSQNLVGETLAHILNGAINRPMRDALLLHQALRESRSGRERSELLISRLVRLHWEPRHLENVKVEFRRRYGERLEEAIAEEILPSSGGSEWGEFCIQLARSSKTHAAKR

**>XP_662412_2_AspE (AN10595)**

MAATATRPRTPGRGQAPIDPPPVPSENGHSQTKDSSRRTSSSLGFLRRSKSTEPIGSKPRGKKMSKAQMEEELRRQREARPKQPPRLPDFSPPPVIETFGGDETRNGVADVTSPLSPSQSRPSRSAMSTPVPPDYSDPYARTESMTHRGRYSYASSYVSTVNNPRRLRRRKDPTPYNILVIGARNSGKTSFLNFLRKSLALPPHKHPSRAPDEVEYDSHNPASEGYTSHYLETEIDGERVGLTLWDSQGLEKNVVDIQLRGVTGFLESKFEETLNEEMKVVRSPGARDTHIHCTFLILDPSRLDENIAAAERAAQGTPRASDSKVLGVLDENFDLQVLRTVIGKTTVVPVISKADTITTAHMAYLRKAVWDSLKKANIDPLEILTLEDQEEYTSSEGEDEEDGETSEAEDAAGETEGGHTKEETEPKAPESPTQRSEGSQQDVGSQAVPLLPFSILSPDKYSLQGDGPIGRKFPWGFADPYNPEHCDFLKLKDSVFSEWRSELREASRVIWYERWRTSRLNRHDAIASPKPRSFGGRTGPDFARRPSGLKHAQNGCHCHIPVSLARPVSFGVPRCQSNWKLSELTVG

**>AN10598 (ANXC4)**

MSLQVNDPRSRGRSRSPSGRTRDRSTSRDPRLPSPGPGPDPARKSGYLLAETVDEKARTRSRSRGASPLRGYRKTSRYDSDSEHEREREREREARDSYTRLRNDRDYYYHSDSGESRGATKRSSQRYSQPPQRSSAQLDAYSDEDIYSDSDDDLAYGDIPGSLERGYYGYKGNSAATRPPSEKPLMTGALNAGTSPRHSAEAVSGYSRYAPGHPARTGPPTSETQSAWAPVPDCEKPGFVPPTSAGDSMPGAFPTTTSGLPTTQYVSSDPVQNPYVQWNTQPPTSGAPYAAPVSAASHQRNPSGDPNLYANPPAFKYAQIDPNVRYSAKPATATTYAPPSKASGQTSDGQYAGVRYTTAPQYSTTATSGSQYVEIAPGSRHTRPASLSVSTNNLSVSGPDPNNPPASPLLEAYKGTYQSISPMPSPILIAPRDDDVSDLEPLDHSTDSERRRRRKSKKSKDEEGGLKEPKSDRSKRGSSRIRHGRHESRDSRGGGPDSVALVSPSTDRRKEVSFYDATDDALALRDALSHSRNIDTKTLIQVLPHLTNHEMLDLRKEYKKHVKIHGKGVNLAKHIRVKLGNSAFGKVCYATALGRWESEAFWANCYYQSGSSRRELLIESLFGRSNGEMREIKESFKDSRYLDSLEKCMKAELKADKFRTAVLLALEEGRQSERDPIDAELVHRDVQALHAALVSRNGGETAMIYIIVRRSDSHLREVLRAYDKIYQRNFARDMIQKSQNLVGETLAHILNGAINRPMRDALLLHQALRESRSGRERSELLISRLVRLHWEPRHLENVKVEFRRRYGERLEEAIAEEILPSSGGSEWGEFCIQLARSSKTHAAKR
