# Supplementary material for: The evolutionary origins and ancestral features of septins
Source: Front Cell Dev Biol. 2024 Jun 26;12:1406966. doi: 10.3389/fcell.2024.1406966 (PMC11238149; doi:10.3389/fcell.2024.1406966)
Supplement: Supplementary file 5 [file Image1.pdf]

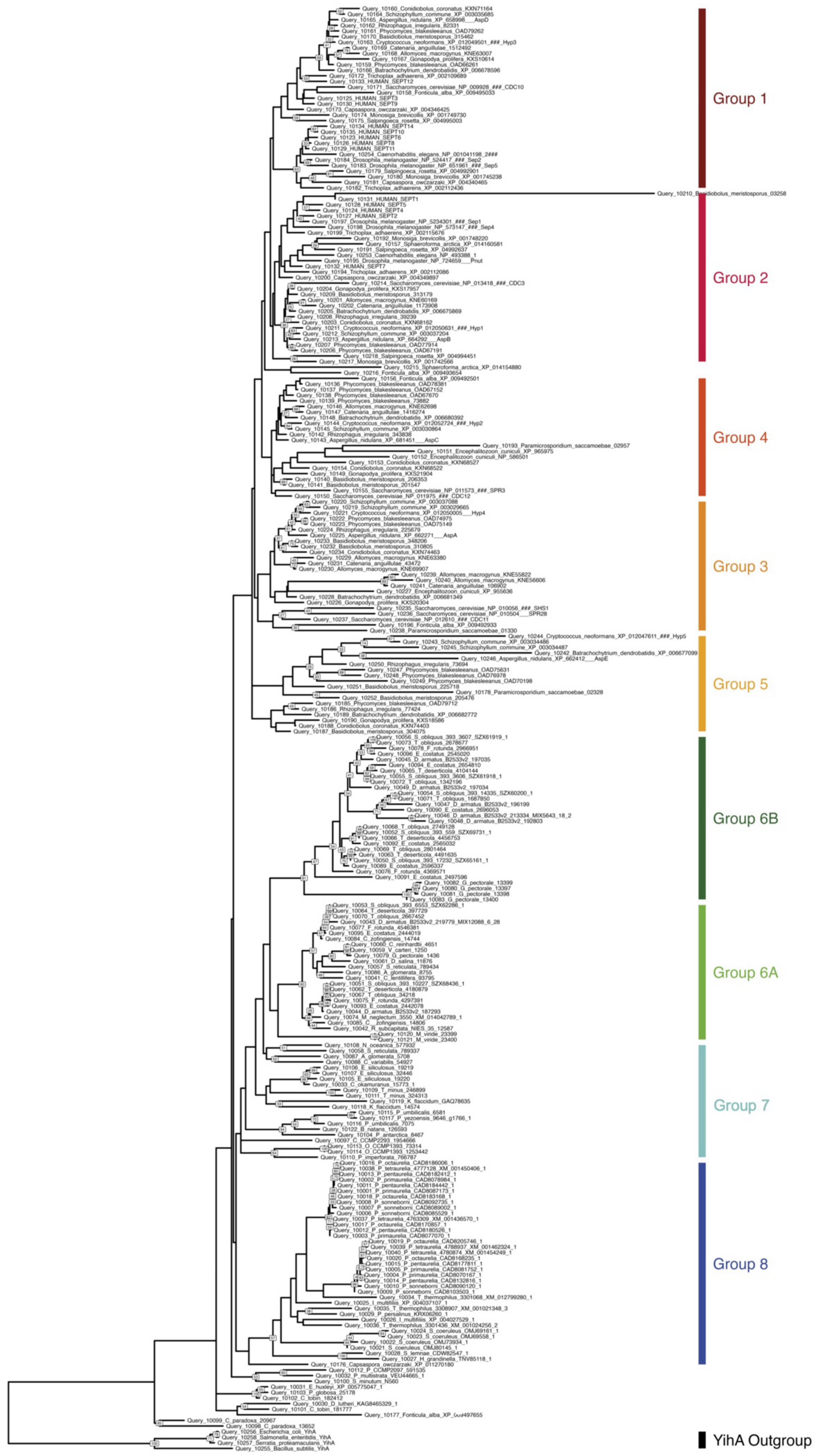

Delic & Shuman et al., Fig. S1

Supplementary Figure 1. RAxML tree of all eukaryotic septins with 1000 bootstraps and YihA family as outgroup. Bootstrap values <25 are not shown. Defined phylogenetic groups are colored and displayed adjacent to tree tips.

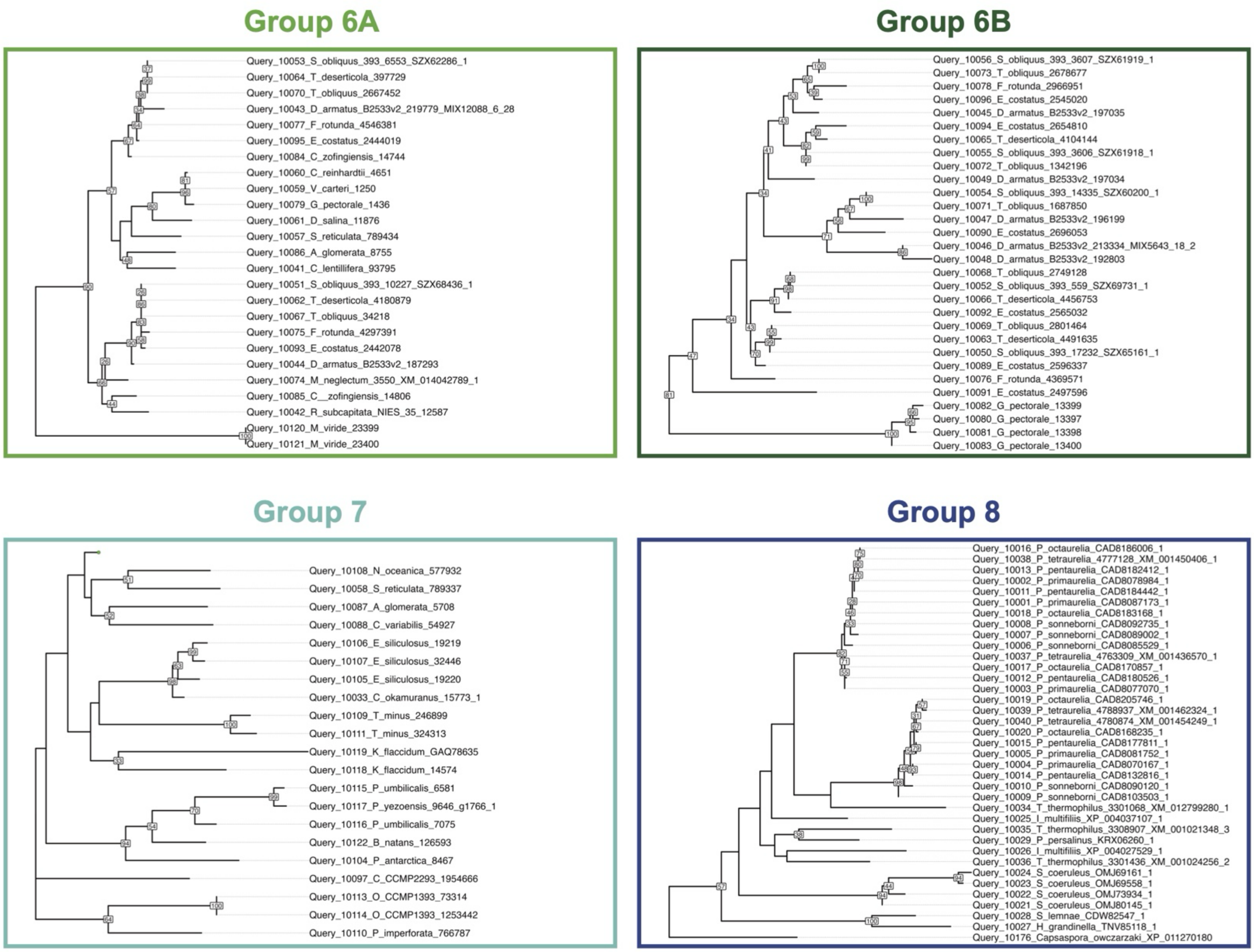

Delic & Shuman et al., Fig. S2

Supplementary Figure 2. Magnified views of septin groups 6-8. The green dot in Group 7 indicates the node for Group 6.

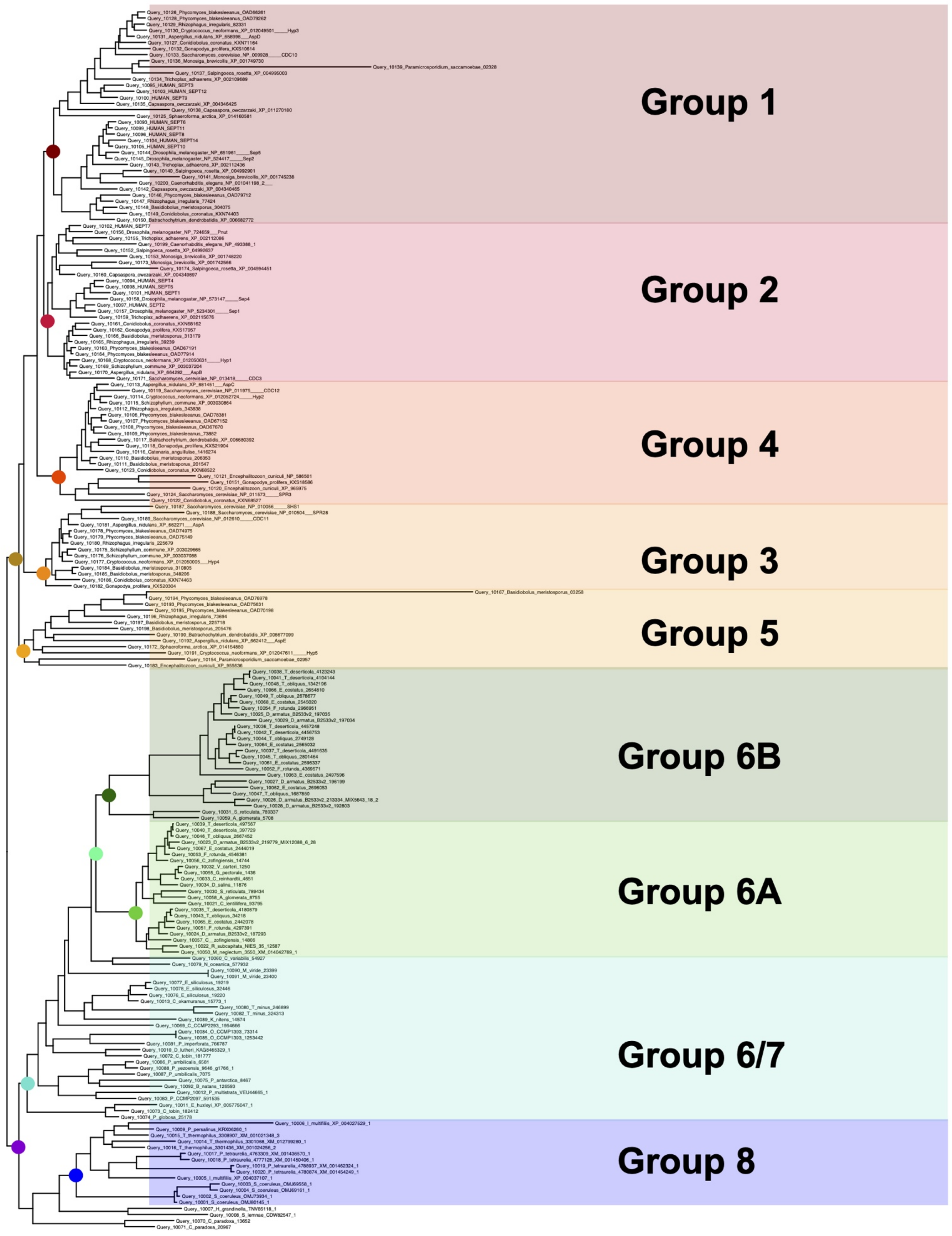

Delic & Shuman et al., Fig. S3

Supplementary Figure 3. IQTree tree 200 septin sequences used in ancestral sequence reconstitution. Groups as defined in Figure 2 are redefined adjacent to branch tips. Colored nodes represent select ancestral sequences used for AlphaFold prediction.

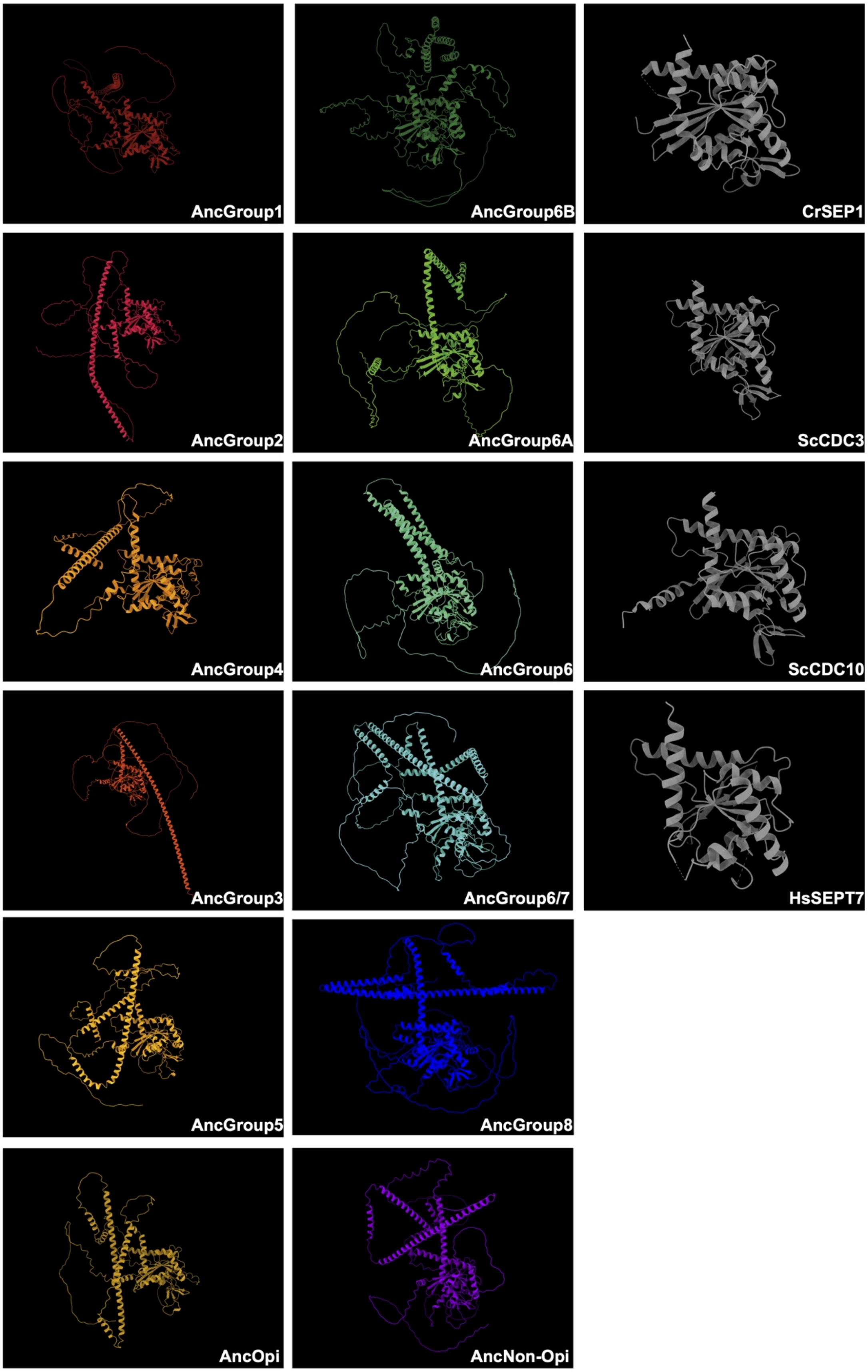

Delic & Shuman et al., Fig. S4

Supplementary Figure 4. AlphaFold-predicted 3D structures of ancestral septins. Structures in grey are experimentally determined Protein Data Bank (PDB) files of septin GTPase domains, included here as references: CrSEP1 (PDB: 5IRR), ScCDC3 (PDB:8SGD), ScCDC10 (PDB:8SGD), HsSEPT7 (PDB:3TW4). Structures are orientated such that the NC-interface is towards the left of the monomer and the G-interface is towards the right.

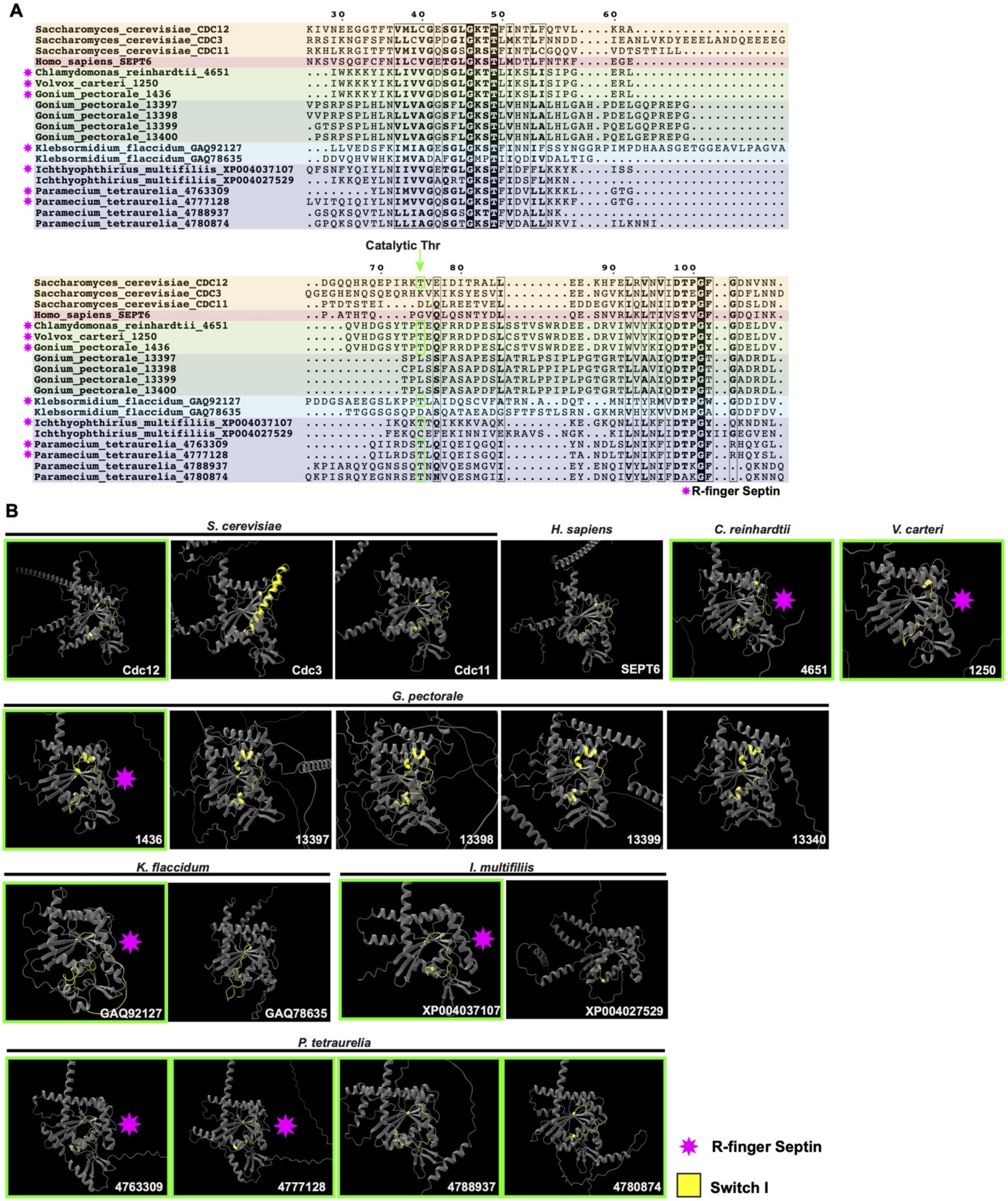

Delic & Shuman et al., Fig. S5

Supplementary Figure 5. Apparent correlation between catalytic threonine in the Switch-I loop and R-finger septins. (A) Multiple-sequence alignment of a subset of representative septins across eukaryotes. Sequences are colored according to their phylogenetic groupings. Regions between between G1 and G3 motifs are shown. Catalytic threonine residues conserved in some septins are indicated by green boxes and with an arrow. R-finger septins are indicated with a magenta star. (B) AlphaFold predicted structures of the septins displayed in (A). Structures are focused on the GTPase domain, with NC-interface towards the left and G-interface towards the right. Switch I region is highlighted in yellow. Green outline, septins with catalytic threonine; magenta star, septins with R-finger.

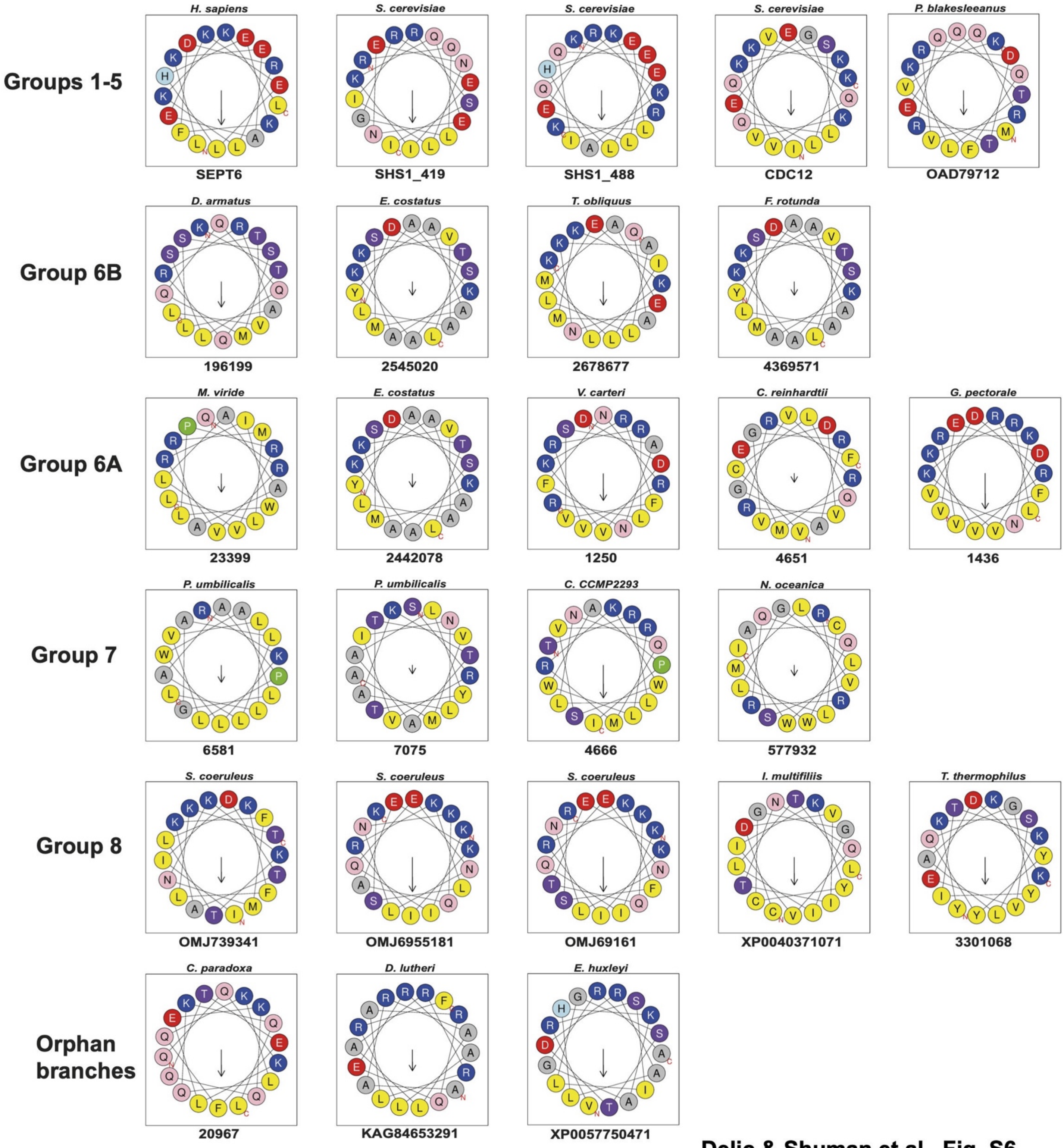

Delic & Shuman et al., Fig. S6

Supplementary Figure 6. Representative helical wheel diagrams of predicted AHs across septin phylogenetic groups. Arrow represents the hydrophobic moment vector. Amino acids are colored according to their chemistry: yellow, hydrophobic; purple, Ser/Thr residues; grey, Gly/Ala residues; blue, basic residues; red, acidic residues; pink, Asp; green, Pro.
